# Carborane Nanomembranes

**DOI:** 10.1021/acsnano.4c16611

**Published:** 2025-02-19

**Authors:** Martha Frey, Julian Picker, Christof Neumann, Jakub Višňák, Jan Macháček, Oleg L. Tok, Petr Bábor, Tomas Base, Andrey Turchanin

**Affiliations:** † Friedrich Schiller University Jena, Institute of Physical Chemistry, Lessingstraße 10, 07743 Jena, Germany; ‡ The Czech Academy of Sciences, Institute of Inorganic Chemistry, 250 68 Husinec-Rez, 1001, Czech Republic; § Department of Chemistry, Middle East Technical University, Ankara 06800, Turkiye; ∥ Central European Institute of Technology (CEITEC), Purkyňova 123, 612 00 Brno-Královo Pole, Czech Republic; ⊥ Center for Energy and Environmental Chemistry Jena (CEEC Jena), Philosophenweg 7a, 07743 Jena, Germany; # Jena Center for Soft Matter (JCSM), Philosophenweg 7, 07743 Jena, Germany

**Keywords:** carboranes, two-dimensional materials, molecular self-assembly, electron irradiation induced chemical synthesis, nanomembranes

## Abstract

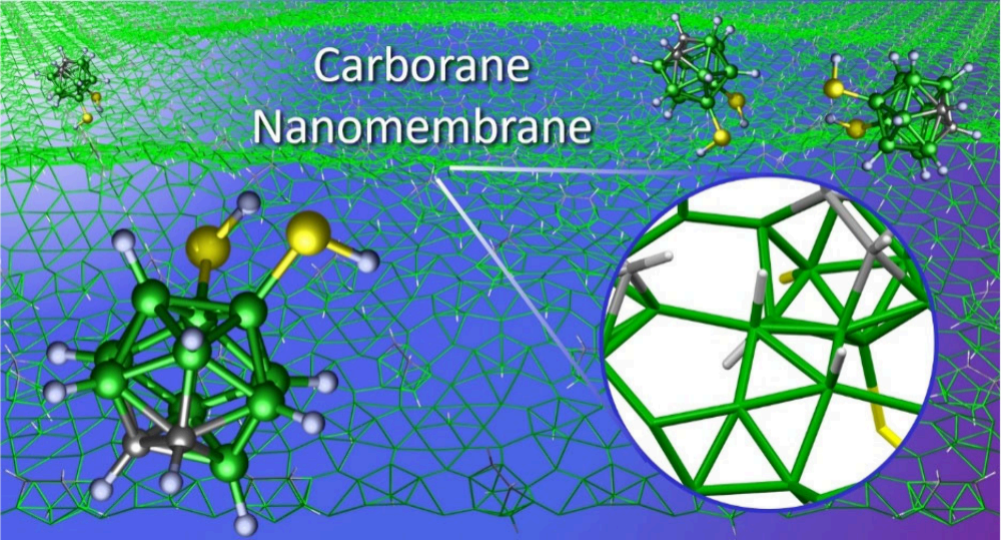

We report on the fabrication of a boron-based two-dimensional (2D) material via electron irradiation-induced cross-linking of carborane self-assembled monolayers (SAMs) on crystalline silver substrates. The SAMs of 1,2-dicarba-*closo*-dodecarborane-9,12-dithiol (**O9,12**) were prepared on flat crystalline silver substrates and irradiated with low-energy electrons, resulting in a 2D nanomembrane. The mechanical stability and compact character of the carborane nanomembrane were improved by using 12-(1′,12′-dicarba-*closo*-dodecarboran-1′-yl)-1,12-dicarba-*closo*-dodecarborane-1-thiol (**1-HS-bis-*p*CB**), a longer, rod-like SAM precursor with two *para*-carborane units linked linearly together. The self-assembly, cross-linking process, and transfer of the resulting membranes onto holey substrates were characterized with different complementary surface-sensitive techniques including X-ray photoelectron spectroscopy (XPS), ultraviolet photoelectron spectroscopy (UPS), and low-energy electron diffraction (LEED) as well as scanning tunneling and electron microscopies (STM, SEM) to provide insight on the structural changes within the cross-linked SAMs. The presented methodology has potential for the development of boron-based 2D materials for applications in electronic and optical devices.

Two-dimensional (2D) cross-linked materials have attracted considerable attention due to their versatile and novel properties resulting from a combination of their chemical composition and size.^1−4^ Their unique properties can largely be attributed to their thickness, which reaches a single atom, as one of the natural limits. The high variability and tunability of the materials’ properties make them interesting for many applications in electronics,^5^ optoelectronics,^6^ energy storage,^7^ ultrafiltration,^8^ and biosensors.^9^ Many inorganic (e.g., graphene,^10^ 2D transition metal dichalcogenides,^11^ boron nitride,^12^ borophene^13,14^) as well as organic (e.g., 2D polymers,^15^ 2D metal–organic frameworks^16^) 2D materials have been previously investigated. The first reported organic 2D material—carbon nanomembranes (CNMs)—were prepared by low-energy electron irradiation induced cross-linking (e-process) of aromatic self-assembled monolayers (SAMs).^17^ During this e-process, the long-range order of the parent SAM is significantly affected; new chemical bonds are formed between the molecules of the original array and the bonds of the binding groups to the metal substrate are partially broken.^18−20^ The functionality of the CNMs can be specifically tailored by selecting an appropriate precursor, resulting in 2D materials with tunable porosity,^18^ thickness,^18,21^ and functional groups.^22−24^ Due to their versatility, CNMs are extensively studied in various application areas such as filtration,^21,25^ electronic and optoelectronic devices,^26,27^ and nanobiotechnology.^28,29^

Functionalized cage molecules are essential constituents of other types of SAMs. These molecules offer distinct advantages due to their symmetry, lower conformational flexibility, and well-defined and adjustable chemical properties.^30,31^ Their small size and shape variations allow precise geometric control and thus control over intermolecular interactions within the respective SAMs. Among the cage molecules, boranes and carboranes have received particular attention due to their rigid and robust three-dimensional structure and chemical and thermal stability. Dicarba-*closo*-dodecaboranes (C_2_B_10_H_12_), possessing almost icosahedral molecular structure, serve as representatives of inorganic molecules within this class. Their chemical properties have been well studied, and various synthetic routes to their functional derivatives have been investigated.^32^ Research on boranes and carboranes in 2D materials has shown their versatility. Early studies explored pristine systems, such as the work on [*closo*-B_12_H_11_S]^3−^ monolayers and a study on carborane-thiol monolayers on gold.^33,34^ Carboranes were also used in nanomachines, like in the study on carborane-wheeled nanocars.^35^ Hybrid monolayers combining organic and carborane components have been developed to leverage the unique properties of carboranes.^36,37^ The first pristine carborane-thiol/thiolate-based SAMs were reported in 2005,^34^ but the fabrication of carborane nanomembranes starting from these monolayers, analogously to CNMs, has not yet been reported.

In addition to their structural and chemical versatility, carboranes have demonstrated varied stabilities under electron irradiation, as highlighted in foundational studies. For example, Hitchcock et al. reported that *closo*-carboranes exhibit remarkable resistance to electron-induced fragmentation compared to their *nido* counterparts, owing to their icosahedral geometry.^38^ However, the potential of carborane-based SAMs for the fabrication of free-standing, cross-linked 2D nanomembranes remained unexplored until now.

Here we demonstrate that carborane-based SAMs can be employed as precursors for the tailored synthesis of free-standing carborane-based 2D materials. For this purpose, SAMs of the carboranethiol derivatives 1,2-dicarba-*closo*-dodecaborane-9,12-dithiol (**O9,12**) and 12-(1′,12′-dicarba-*closo*-dodecarboran-1′-yl)-1,12-dicarba-*closo*-dodecarborane-1-thiol (**1-HS-bis-*p*CB**) were prepared on flat crystalline silver substrates and subsequently irradiated with low-energy electrons to induce their lateral cross-linking, Figure 1. The formation of SAMs as well as the changes induced by the electron irradiation were studied both experimentally using X-ray (XPS) and ultraviolet photoelectron spectroscopies (UPS), scanning tunneling microscopy (STM), low-energy electron diffraction (LEED) and time-of-flight secondary ion mass spectrometry and computationally using density functional theory (DFT). The successfully cross-linked monolayers were transferred onto rigid and holey substrates showing the formation of nanomembranes which were further characterized using optical and scanning electron microscopy (SEM).

**Figure 1:**
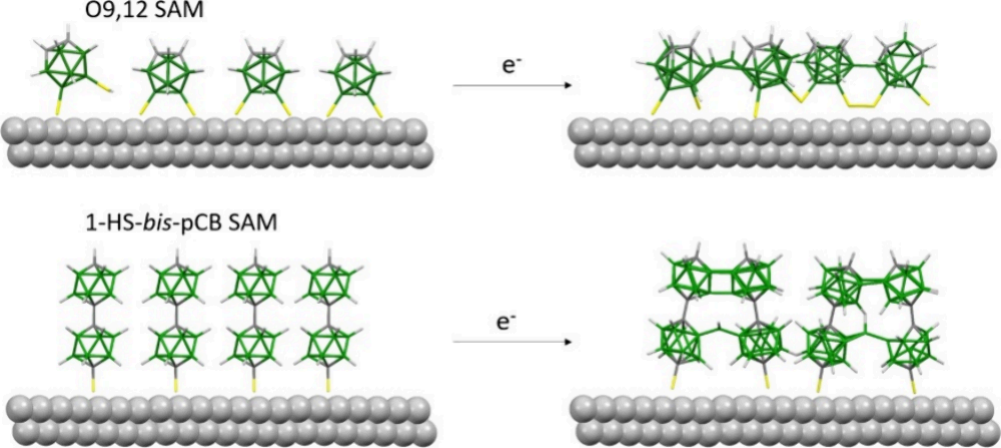
Schematic representation of the cross-linking process based on DFT calculations (see Supporting Information (SI) for details). Initially ordered carborane SAMs are converted into short-range-ordered membranes using low-energy electron irradiation.

## Results and Discussion

### Pristine SAMs on Ag(111)

**Figure 2:**
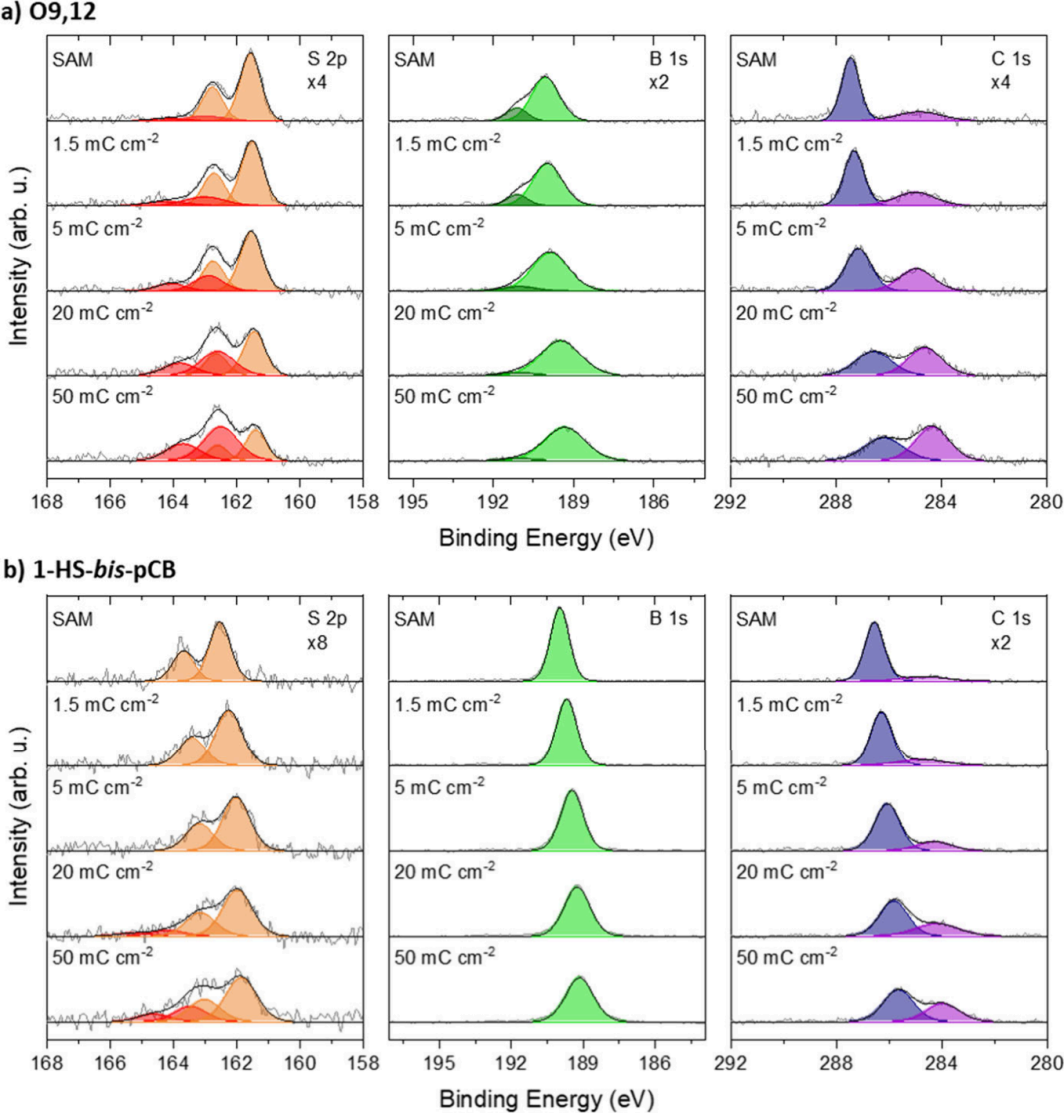
S 2p, C 1s, and B 1s XP spectra of a) **O9,12** and b) **1-HS-bis-*p*CB** SAMs stepwise cross-linked into a nanomembrane *via* electron irradiation with an energy of 50 eV. The spectra’s intensities have been multiplied by the indicated factor for better representation.

The SAMs of the respective carborane derivatives, either **O9,12** or **1-HS-bis-*p*CB**, were prepared on Ag(111) substrates by physical vapor deposition (PVD) in ultrahigh vacuum (UHV). The *in situ* mass spectrometry measurements were applied to control the molecular integrity during deposition (Figure S1). The successful formation of SAMs was then verified by XPS. High-resolution XP spectra of the S 2p, C 1s and B 1s core level electrons of the respective SAMs are shown in Figure 2 (**O9,12** (a) and **1-HS-bis-*p*CB** (b)). The S 2p spectrum of the **O9,12** SAM reveals two distinct sulfur species. The first doublet, with binding energies (BEs) of 161.6 eV (S 2p_3/2_) and 162.8 eV (S 2p_1/2_), corresponds to the formation of thiolates (orange) on the silver substrate. Similar BEs have been observed for **O9,12** SAMs prepared from a solution on gold substrates at ambient conditions,^31^ confirming the successful formation of the SAM. The second doublet with the BE values of 163.0 eV (S 2p_3/2_) and 164.2 eV (S 2p_1/2_), respectively, can be attributed to undissociated thiol groups (red).^20,39^ Each molecule of **O9,12** contains two thiol groups in adjacent positions and can be present in SAMs as either singly (monovalent) or doubly (divalent) bound to the metal substrate.^31^ The presence of the thiol species is explained by the monovalent binding mode, where only one thiol group of the molecule undergoes deprotonation. Approximately, three out of four molecules exhibit divalent binding, as determined by the area ratio of the two sulfur species, with slight variations influenced by deposition conditions (Table S1). For the **1-HS-bis-*p*CB** SAM, only one sulfur species was observed with BE values of 162.5 eV (S 2p_3/2_) and 163.7 eV (S 2p_1/2_), corresponding to thiolates. This behavior is expected since **1-HS-bis-*p*CB** contains only one thiol group and does not exhibit different binding modes under these experimental conditions. Notably, the BE of the thiolates in **1-HS-bis-*p*CB** is shifted to higher values by 0.9 eV compared to **O9,12**. This difference is caused by sulfur attached to an electron-donating boron vertex in **O9,12**, but to an electron-accepting carbon vertex in **1-HS-bis-*p*CB**. As a result, the sulfur atoms in **O9,12** are more negatively charged than those in **1-HS-bis-*p*CB**. The B 1s signal of the **O9,12** SAM exhibits two peaks: one at 190.0 eV (green) and the other at 191.1 eV (dark green). The shoulder peak observed at the higher BE corresponds to the two boron atoms bound to sulfur, while the dominant peak at the lower BE value corresponds to the remaining eight boron atoms. The higher BEs for the two boron atoms bound to sulfur are consistent with the electron withdrawing effect of sulfur. The ratio of the two components is (1.0:4.4) ± 0.4 (Table S2), which is close to the expected nominal ratio of 1:4. In contrast, the thiol group in the **1-HS-bis-*p*CB** SAM is bound to the carbon atom of the carborane cage, leaving all boron atoms virtually identical, resulting in a single narrow (full width at half maximum, fwhm: 0.9 eV) peak at 190.0 eV in the B 1s spectrum. The C 1s signal of the **O9,12** SAM exhibits one main component at a BE of 287.4 eV (blue), which is attributed to the two carbon atoms of the carborane molecule. A small shoulder accompanying the main peak at 284.8 eV is typical of C–C/C–H bonds originating from minor hydrocarbon impurities (violet). In the **1-HS-bis-*p*CB** SAM, the carborane carbon peak shifts by 0.9 eV to a lower BE value due to different atomic neighborhoods: In **1-HS-bis-*p*CB**, the neighboring atoms of carbon are exclusively boron, while in **O9,12** one neighboring atom is carbon. Consequently, the **1-HS-bis-*p*CB** carbon carries a lower positive partial charge than its **O9,12** counterpart.

The effective thickness of the **O9,12** SAM is determined to be 5 ± 1 Å based on the attenuation of the Ag 3d_5/2_ signal, which matches the size of a single molecule (approximately 6 Å). Considering the nearest neighbor distance between two molecules of about 7.7 Å, measured with STM (*vide infra*), which contributes to the reduction of the average film thickness, the experimentally determined value corresponds to a complete, densely packed monolayer. Similarly, for **1-HS-bis-*p*CB**, the SAM thickness is measured to be 10 ± 2 Å, which is twice as thick as the value measured for **O9,12** SAM. The difference fits the molecular structure of **1-HS-bis-*p*CB**, which consists of two carborane cages linked by a single bond and standing upright on the surface. The elemental S/C/B stoichiometry ratio of the **O9,12** SAM is found to be (1.0 ± 0.2):(1.5 ± 0.3):5, closely matching the nominal one (1:1:5). In the case of the **1-HS-bis-*p*CB** SAM, the elemental S:C:B ratio of (3.9 ± 0.7):(0.6 ± 0.1):20 also shows very good agreement with the nominal molecular stoichiometry of 4:1:20. Sulfur atoms appear with a slightly lower intensity due to the shielding by the carborane cage molecules above them. These experimental results are in excellent agreement with the expected stoichiometry and film thickness, and the high quality of the prepared SAMs is further confirmed by the absence of oxygen in the samples (Figure S2).

**Figure 3:**
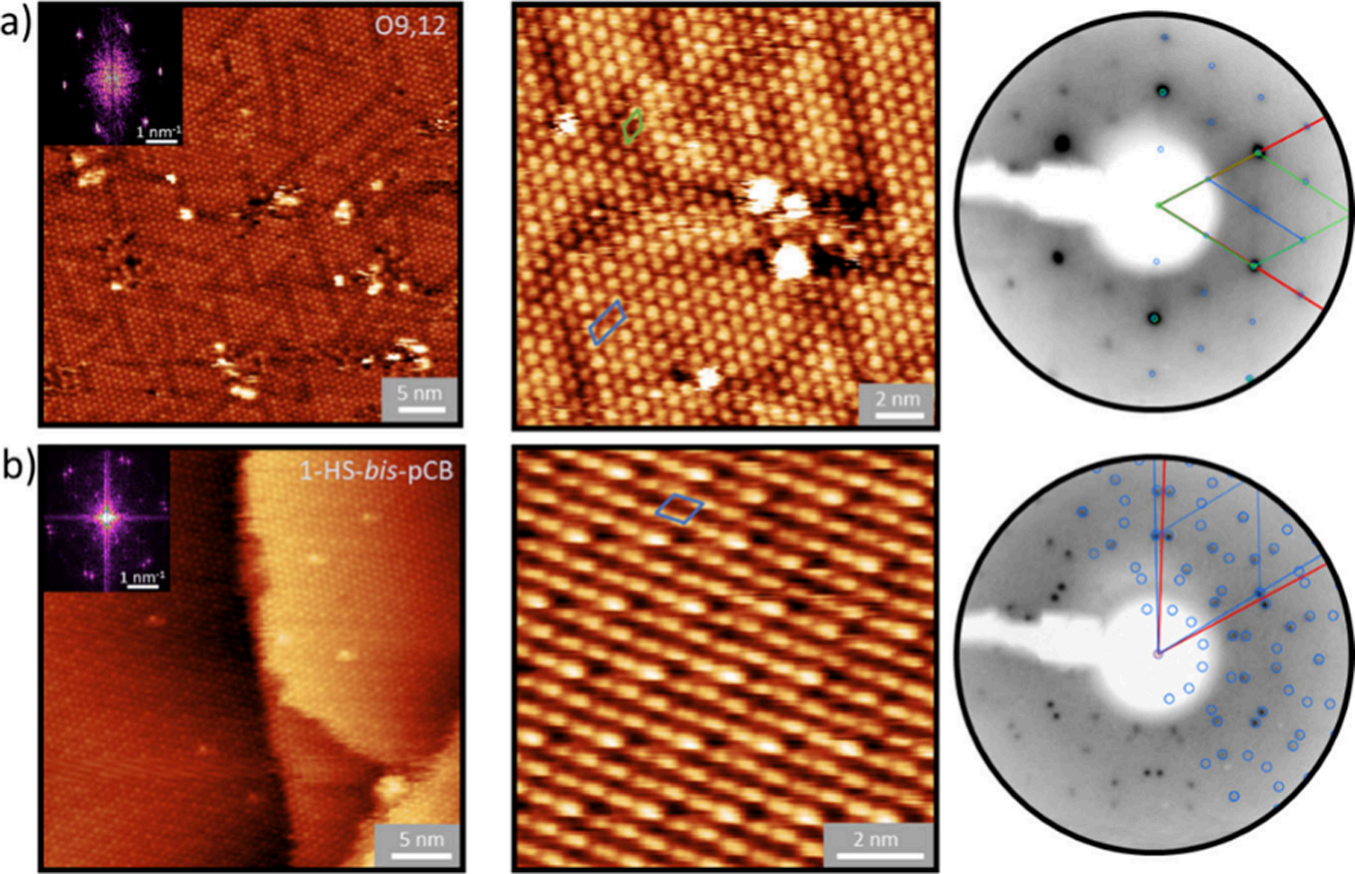
STM and LEED data of a) **O9,12** and b) **1-HS-bis-*p*CB** SAMs on Ag(111). Simulated LEED structures of the **O9,12** SAM are highlighted in green (one molecule) and blue (two molecules) and of **1-HS-bis-*p*CB** SAM is highlighted in blue (one molecule). (Conditions: a) 45 × 45 nm^2^, 1.5 nA, 0.1 V; 18 × 18 nm^2^, 0.5 nA, 0.1 V; 34 eV; b) 35 × 35 nm^2^, 0.5 nA, 1.4 V; 10 × 10 nm^2^, 0.5 nA, 1.4 V; 36 eV).

The structural arrangement of the **O9,12** and **1-HS-bis-*p*CB** molecules on the Ag(111) substrate was investigated using STM and LEED (Figure 3). Both SAMs exhibit a highly ordered arrangement characterized by hexagonal symmetry. The results are summarized in Table 1. Figure 3a,b show the LEED pattern of the **O9,12** SAM obtained at an electron energy of 34 eV together with two simulated reciprocal lattices. The LEED pattern corresponds to a unit cell consisting of a single molecule, marked in green in Figure 3a, with the parameters of a_1_ = (7.7 ± 0.1) Å, a_2_ = (7.8 ± 0.2) Å, and the angle between the two vectors of (119.6 ± 0.4)°. Notably, the lattice vectors of the molecular structure align with the vectors of the Ag(111) substrate, which is in contrast to the previously reported structure of **O9,12** SAM on Au(111).^31^

**Table 1:** Lattice Parameters of Quantitative LEED Analysis

Structure	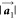 [Å]	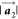 [Å]	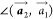 [deg]	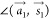 [deg]	*M̑*
**O9,12(two molecules)**	**7.7(1)**	**15.5(2)**	**119.6(4)**	**0.0(4)**	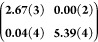
O9,12 (HOC structure)	7.705	15.603	120.00	0.00	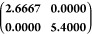
**O9,12(one molecule)**	**7.7(1)**	**7.8(2)**	**119.6(4)**	**0.0(4)**	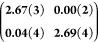
**1-HS-bis**-***p*****BC**	**6.85(1)**	**6.85(1)**	**120.02(6)**	**3.04(5)**	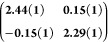
1-HS-bis-*p*BC (HOC structure)	6.819	6.819	120.00	3.00	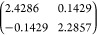

However, the single-molecule unit cell (green in Figure 3a) does not explain the weaker spots observed in the LEED pattern. Therefore, a new structure covering three rotational domains was constructed to fit all of the LEED spots in the LEED pattern. The unit cell of this structure, with the lattice parameters given inTable 1, is highlighted in blue. The second lattice vector of 15.5 (±0.2) Å is twice as long as that in the green structure. The epitaxy matrix which describes the structural relation between substrate and adsorbate is measured to be . Since the values of M̑ are only slightly out of a higher order commensurate (HOC) structure (see Table 1), we conclude that the **O9,12** molecules are most likely arranged in a HOC structure on Ag(111) with two molecules per unit cell.

Furthermore, real space STM investigation of the **O9,12** SAM on Ag(111) shows a highly ordered hexagonal structure over large scales where each dot represents one molecule (Figure 3a). At first glance, the presence of two distinct species of **O9,12** molecules is observed. The predominant molecular species appears with a brighter contrast (corresponding to a higher STM height) and alternates with the minor molecular species, which exhibits a darker contrast (corresponding to a lower STM height), forming rows of two molecules along the high symmetry directions of Ag(111). The STM height difference of both species is measured from line scans as 22 ± 6 pm (Figure S3, green line). This height difference matches the height difference of simulated STM images of two **O9,12** molecule species on Au(111) reported in the literature.^31^ Accordingly, we attribute these two distinct molecular species on Ag(111) to the two previously mentioned binding modes: monovalent molecules with one thiol and one thiolate group and divalent molecules with both groups bound to the Ag(111) substrate as thiolates. Unlike the **O9,12** SAM on Au(111), where monovalent bonds dominate,^31^ the divalent molecules (i.e., those with the larger apparent height under STM conditions) predominate on the Ag(111) substrate. The ratio of monovalent to divalent **O9,12** molecules, as estimated from STM images, is approximately 1:3 which aligns closely with our XPS data.

Additional analysis of the STM images shown in Figures 3a and S3 provides further valuable insights. Along the blue line scan in Figure S3, the STM height difference of ∼12 pm is observed between neighboring molecules, whereas such a difference is not observed along the other lattice vector directions (red line scan). Considering the combined results from LEED and STM, it can be concluded that the blue unit cell consists of two molecules with varying adsorption heights.

The LEED pattern of the **1-HS-bis-*p*CB** SAM (Figure 3b) exhibits an even more complex arrangement. By including multiple scattering up to fourth order, mirror, and rotational domains, we found a structure that explains all LEED spots. The adsorbate lattice vectors have a length of a_1_ = a_2_ = 6.85 (±0.01) Å, an enclosing angle of 120.02 (±0.06)°, and an angle between the first adsorbate lattice vector () and the first substrate lattice vector () of 3.04 (±0.05)°.

We also measured STM images with a molecular resolution (Figure 3b). In the large scale STM image, we observe two different mirror domains predicted by LEED that are clearly highlighted in the fast Fourier transform (FFT) image and in Figure S4. They can be attributed to a unit cell consisting of one molecule (blue in Figure 3b). In addition, molecules with a higher STM height are visible in the close-up STM image. Quantified FFT analysis provided the epitaxy matrix between this superstructure (green in Figure S5a and b) and the single molecule unit cell to be  (Figure S5b). Since we know the relation between the one molecular structure and the Ag(111) from LEED very well, the epitaxy matrix between the superstructure and Ag(111) is
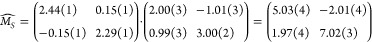
This corresponds to a commensurate structure consisting of 7 molecules (Table 1). Figure S6b shows the real space representation of the commensurate structure (green) and the Ag(111) substrate (red). The lattice points of the unit cell consisting of 7 molecules are aligned with those of Ag(111). We conclude that the larger apparent height of some of the molecules in the STM images (Figure 3b) is due to them being on top of silver atoms, while other molecules may occupy lower-height surface sites.

Summarizing the XPS, STM, and LEED data, it can be concluded that both derivatives, **O9,12** and **1-HS-bis-*p*CB**, form densely packed and well-ordered monolayers on Ag(111) substrates.

### Lateral Cross-linking via Electron Irradiation

In the next step, the carborane SAMs were stepwise converted into nanomembranes via low-energy electron irradiation induced cross-linking and characterized using XPS, UPS, LEED, STM, and time-of-flight secondary ion mass spectrometry (ToF-SIMS). Figure 2 shows selected XP spectra after irradiation doses of 1.5 mC/cm^2^, 5 mC/cm^2^, 20 mC/cm^2^ and 50 mC/cm^2^. In the S 2p spectrum of both molecules, the intensity of the doublet corresponding to the thiolate sulfur atoms (orange) decreases as a direct consequence of irradiation. Conversely, the component at a higher BE (red) of 163.0 eV (S 2p_3/2_) and 164.2 eV (S 2p_1/2_), respectively, increases, indicating that the thiolate bonds of the molecule to the silver substrate are broken during electron irradiation followed by the formation of disulfide bridges between adjacent molecules. The proportion of disulfide moieties gradually increases during irradiation, reaching 63% of the total sulfur content for **O9,12** and 27% for **1-HS-bis-*p*CB** (Figure 2, Table S1, and Figure S7). Upon electron irradiation, the B 1s signal broadens, with the fwhm increasing from 1.2 to 1.8 eV for **O9,12** and from 0.9 to 1.4 eV for **1-HS-bis-*p*CB**. This indicates the formation of new boron species resulting from the cross-linking of adjacent carborane molecules into a 2D nanomembrane. This phenomenon is similar to that observed in the C 1s XP spectra of organic aromatic SAMs during electron-irradiation induced formation of CNMs.^18^ In addition, the B 1s signal shifts to a lower BE value (Table S1), as the structure and dipole moment of the molecules are affected by electron irradiation and subsequent breaking of the sulfur–silver bonds. The cross-linking of the carborane molecules also induced changes in the respective C 1s spectra. The C 1s component assigned to carbon atoms in the **O9,12** SAM spectrum is broadened, with the fwhm increasing from 0.8 to 1.8 eV. The signal gradually shifts down from 287.4 to 286.1 eV, indicating the formation of new chemical states and new types of bonds, as supported by computational analysis (discussed in more detail later in this study). The minor component at 284.8 eV in the C 1s spectrum of the **O9,12** SAM increases to ∼55% of the total carbon intensity. This is attributed to the formation of new C–C and C–B bonds (Figure S7), which are more comparable to the character of carbon atoms in hydrocarbons rather than any additional deposition of carbonaceous impurities. No increase in the total carbon content was observed. The hypothesis that these additional carbon species arise from C–C bonds with nearest neighbor molecules is further supported by the absence of oxygen in the sample, even after irradiation (Figure S2). Similar effects are observed for the C 1s spectra of **1-HS-bis-*p*CB**, where the fwhm increases from 0.9 to 1.5 eV, and the BE shifts from 286.5 to 285.6 eV. The proportion of carbon atoms typical for hydrocarbons increases from 14% to 38%. The thickness of nanomembranes prepared from **O9,12** molecules remains at 5 ± 1 Å, indicating superior stability during the irradiation. The elemental C:S:B ratio of the cross-linked membrane is determined to be (1.3 ± 0.3):(1.9 ± 0.4):5. A comparison with the nominal stoichiometry of the **O9,12** molecule (1:1:5) suggests that some minor amount, consisting mainly of the uppermost carbon and boron atoms of the monolayer, desorbed during the process. The original attenuation of the sulfur signal from the thiol groups near the metal surface (underneath the carborane backbones) is reduced, resulting in a higher apparent intensity of the S 2p signal. Congruently, the effective thickness of the **1-HS-bis-*p*CB** layer decreases from 10 ± 1 Å to 9 ± 1 Å upon irradiation. The elemental composition after irradiation is determined to be C:S:B in a ratio of (3.2 ± 0.6):(0.8 ± 0.2):10, also indicating some decrease in the percentage of boron, likely due to desorption.

**Figure 4:**
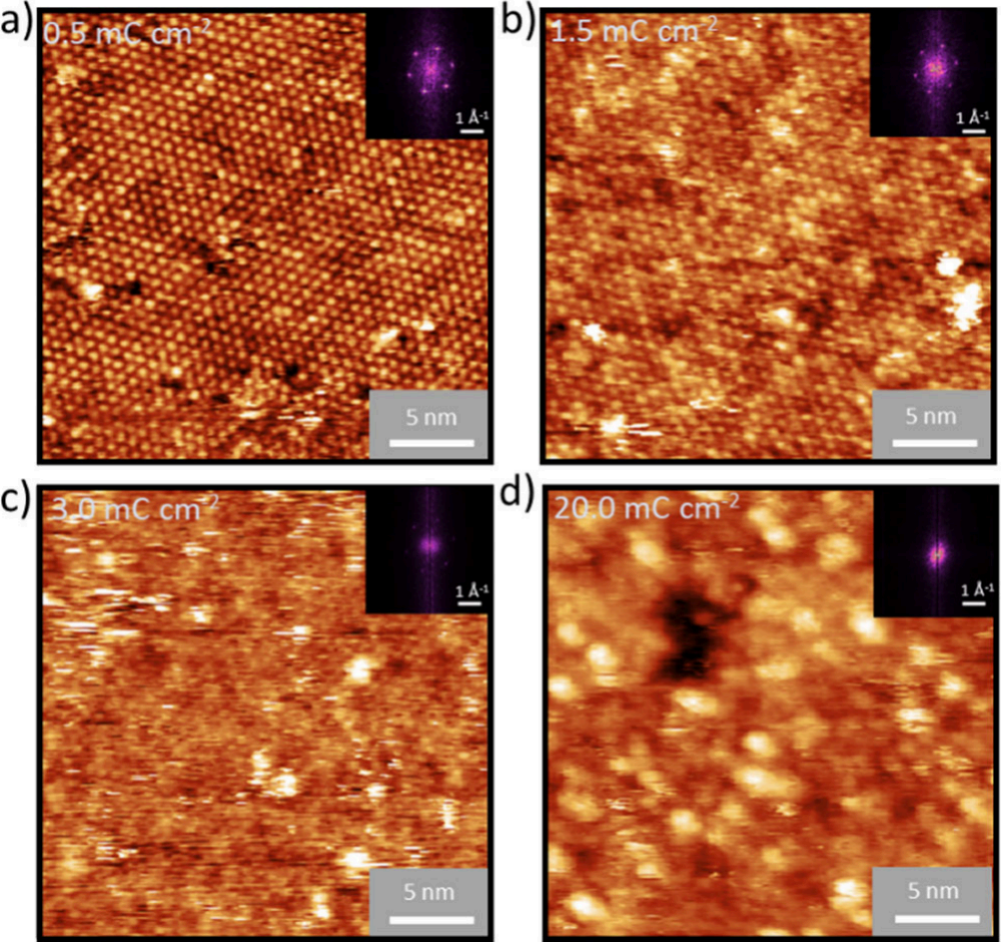
STM images of electron-irradiated **O9,12** SAMs on Ag(111) with electron doses of a) 0.5, b) 1.5, c) 3.0, and d) 20.0 mC cm^–2^ (Conditions: 27 × 27 nm^2^, 1.0 nA, 0.1 V).

The structural changes in **O9,12** SAM induced by electron irradiation were investigated by using LEED (Figure S8). During electron irradiation, the molecules undergo cross-linking, and their positions change due to the formation of new bonds, resulting in increased surface corrugation. This transformation is manifested by a loss of long-range order, which is characteristic of the initially well-organized SAM. This leads to a decrease in the intensity of the LEED spots and their eventual disappearance. After the first irradiation step (0.5 mC/cm^2^), the weaker LEED spots disappear, leaving only the spots corresponding to the green structure in Figure 3. At an irradiation dose of 3.0 mC/cm^2^, no LEED spots are observed, indicating complete disruption of the long-range order. The same process can also be observed in real space on a smaller lateral scale using STM. After an irradiation dose of 0.5 mC/cm^2^ (Figure 4a), most of the molecules still retain a highly ordered structure. However, the distinction between the mono- and divalent binding modes becomes more challenging, and the rearrangement of **O9,12** molecules within the SAM begins. After further irradiation to 1.5 mC/cm^2^, the two binding modes are indistinguishable (Figure 4b). In addition, the cross-linking process leads to an increase in the number of adsorbates on the substrate of the molecular layer. At the same time, the periodic ordering of the molecules decreases, as is evident from changes in the FFTs of the STM images (Figure 4a-d). This trend continues with further irradiation up to 3.0 mC/cm^2^, where, in contrast to LEED, the FFT spots can still be discerned (Figure S8), indicating the presence of a locally ordered structure on the nanometer scale. At an electron dose of 20 mC/cm^2^, the original molecules are no longer observed in the STM images, and the formation of pores (voids) with an edge size of up to a few nanometers appears (Figure 4d). These pore sizes resemble a similar phenomenon observed in CNMs on Au(111) with the size of 0.5 nm.^40^ The formation of pores as well as potential cracks in the cross-linked membranes can be attributed to the formation of new bonds that bring the backbones of the original carborane clusters closer together, where new bonds are formed, or further apart, depending also on the extent to which these clusters open up into 2D-polyhedral networks. The occurrence of broken bonds and the formation of new bonds among the original molecules of the SAM upon irradiation agree well with the changes observed in the XP spectra. Once the SAM is fully cross-linked, the STM images show no further evidence of changes, which is consistent with the results of LEED analysis (Figure S8).

ToF-SIMS analysis was done with the SAM of **O9,12** and the respective cross-linked nanomembrane (Figure S9). The spectra of the starting SAM exhibit a strong fragment of M_1_Ag_3_ (M: C_2_B_10_S_2_) with typical isotopic envelopes corresponding to the calculated one (Figure S9a).^41^ This typical fragment continuously disappears as a consequence of electron-induced irradiation, which fits the loss of long-range order documented well by STM and LEED (Figure S9b-c). Within the cross-linking, molecules can connect in various different ways, as addressed computationally, and form a polymer-like entity. This polymer character contains potentially many different fragments, each one of which has a concentration lower than is the detection limit of the method. The disappearance of a strong fragment has therefore not been accompanied by the rise of any new fragments. Furthermore, the changes observed in the XP spectra due to cross-linking (such as broadening of the B 1s signal and increase of sulfur typical of a disulfide in the S 2p region) fit very well the variety of computationally optimized cross-linking motifs.

Obtaining atomically precise information on the rearrangement of molecules due to their electron-induced cross-linking is experimentally very challenging. We have therefore carried out a DFT computational investigation aimed at providing initial insight into different local structural features that might form in the cross-linked carborane nanomembranes.

Figure S10 illustrates the various basic motifs found in the optimized structures. Simple cross-linking through single B–B, and bridging μB-*H*-B and μ-H bonds (Figure S10a) results in the formation of four-, five-, and six-membered rings that connect two or three molecules. This brings the molecules close together compared with their positions in the original SAM arrays. Besides that, a single boron atom or BH moiety can be pulled out of one cluster to act as a bridge to the adjacent one, introducing another type of connecting motif. It is worth noting that all of these structural features have been observed in various borane and carborane cluster species.^42^ Also observed was progressive cage opening (Figure S10h) that leads to the formation of two-dimensional deltahedral bridges, as schematically shown in Figure S10i,j. The diversity of all these motifs fit very well the loss of long-range order observed both in LEED as well as in STM measurements (Figures 2 and 4), or using also the method of ToF-SIMS (Figure S9). In addition to new bonds on the level of the carborane clusters, the computational investigation also revealed changes that involve the anchoring thiol groups and specifically their transformation from thiolates to disulfides, which is in very good agreement with the results of XPS discussed earlier (Figure 2).

The electronic structure of both **O9,12** and **1-HS-bis-*p*CB** SAMs before (red) and at various levels of cross-linking, i.e. after electron irradiation at doses of 5 mC/cm^2^ (violet) and 50 mC/cm^2^ (blue), were analyzed using ultraviolet photoelectron spectroscopy (UPS). Figure S11a shows the UP spectrum of the **O9,12** SAM (red curve), which exhibits peaks at 8.2, 6.0 and 5.4 eV. The signal at 5.4 eV can be attributed to the silver substrate and is also observed in the spectrum of the **1-HS-bis-*p*CB** SAM in Figure S11b. The other two signals can be attributed to the **O9,12** SAM and decrease in intensity upon irradiation. Similarly, the spectra of **1-HS-bis-*p*CB** show a signal at 7.6 eV that decreases with irradiation.

The work function of **O9,12** SAM increases from 3.5 ± 0.1 eV to 4.3 ± 0.1 eV during irradiation, while the value of 4.6 ± 0.1 eV was measured for a bare Ag(111) substrate. For **1-HS-bis-*p*CB**, the work function increases from 4.0 ± 0.1 eV to 5.3 ± 0.1 eV, exceeding the work function value typical for the silver substrate after the initial irradiation step of 5 mC/cm^2^. **O9,12** is a small molecule with a strong dipole moment (5.7 D)^41,43^ mainly due to the positioning of the two carbon atoms in its cage and the two sulfur atoms.^44^ In the **O9,12** SAM, the carbon atoms are oriented away from the silver substrate and the dipole has its negative pole close to the substrate, thus lowering the work function value. During irradiation, there are several aspects that potentially change the work function value. In addition to changes within the molecule caused by general reorganization including the removal of some of the carbon atoms, as experimentally indicated by XPS, the thiolate bonds of **O9,12** to the silver substrate are also broken, and the effect of the molecule, representing originally a relatively strong molecular dipole, on the surface potential is reduced. In contrast, the **1-HS-bis-*p*CB** molecule has a significantly lower inherent dipole moment compared to **O9,12**, and, in its SAM, the work function changes are more associated with the effect of the thiolate sulfur bond to the silver substrate.^45^ During electron irradiation, the **1-HS-bis-*p*CB** molecules are then modified primarily at the top, which is directly exposed to the electron beam. The part closest to the Ag substrate retains its original arrangement for longer while the more distant parts from the substrate are either removed or rearranged into more open structures. The rearranged carbon atoms farthest away from the substrate lose their positive charge, while those closest to the substrate retain it. The result is an increase in the polarization of the layer in the direction toward the metal substrate and thus an increase of its work function value.

We tested the thermal stability of the irradiated **O9,12** samples and annealed them in UHV (Figure S11). We found that the cross-linked carborane SAMs are thermally stable up to ∼300 °C. Above this temperature, modifications of the carbon and boron bonds were found leading to chemical changes in the obtained membrane. A more detailed analysis is presented in the SI.

### Freestanding Carborane Nanomembranes

**Figure 5:**
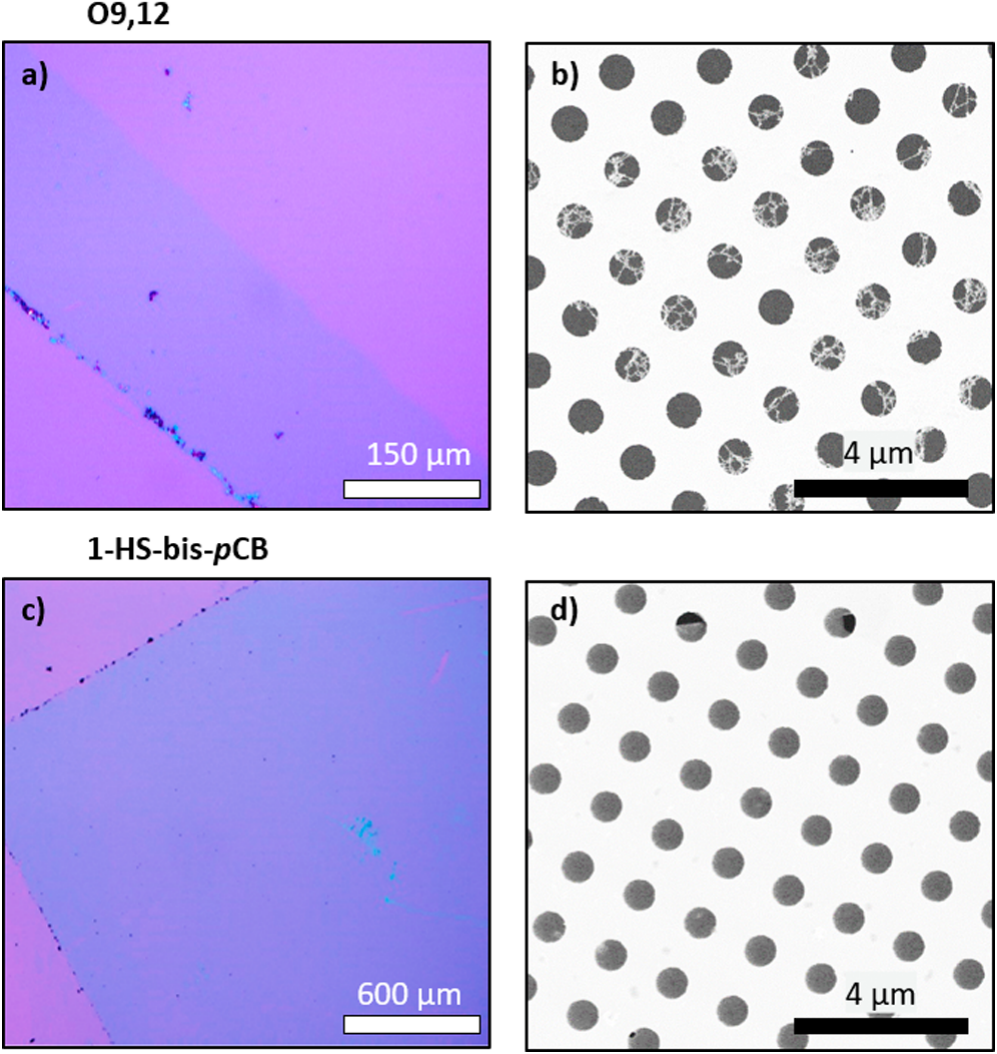
a) Optical microscopy image of a transferred **O9,12**-based membrane onto a silicon/silicon oxide wafer. b) Scanning electron micrograph of the carborane membrane placed on a TEM grid. c, d) Optical microscopy and SEM images of a membrane fabricated from **1-HS-bis-*p*CB** as the precursor.

Finally, the cross-linked carborane SAMs on Ag/Mica substrates were transferred onto SiO_2_/Si substrates and transmission electron microscope (TEM) grids using a PMMA-based transfer method previously established for CNMs (see Methods and Experimental Details).^46^ The resulting membranes are characterized by optical microscopy and scanning electron microscopy (SEM). In the optical microscopy images (Figure 5a,c), the transferred membranes (blue) are visible on the silicon oxide wafer due to interference effects. XPS analysis reveals the presence of B 1s and S 2p signals in this region (Figure S13), confirming the successful cross-linking of the carborane molecules by low-energy electron irradiation and the formation of mechanically stable membranes of molecular thickness. Especially the transferred carborane-based nanomembrane prepared from **1-HS-bis-*p*CB** molecules shows large regions of several square millimeters without any macroscopic defects. The presence of residual sulfur confirms that some of the binding groups are still connected to the cross-linked carborane cages, which can be used for further functionalization of the prepared nanomembranes. Using SAM precursors based on carboxylic-type of binding groups, the residual sulfur could possibly be avoided, enlarging the range of applications even more.^47^

To investigate the characteristics of the formed material in more detail, SEM analysis was performed after both nanomembranes were transferred onto TEM grids. The nanomembrane prepared from **O9,12** molecules shows a free-standing reticulated structure with holes and cracks (Figure 5b), suggesting that the low thickness of 5 Å is not sufficient to form a continuous membrane. In contrast, the longer **1-HS-bis-*p*CB** molecule enables the formation of stable and continuous nanomembranes as shown in Figure 5d. Here, most of the TEM grid holes are completely covered by the respective nanomembrane without any visible defects. This shows great potential of these molecules and of the presented fabrication method to prepare and tailor the properties of carborane-based nanomembranes as there is a large variety of borane and carborane cage molecules differing in their chemical composition, size, and structure that can be specifically designed for these nanomebranes.

## Conclusion

In this contribution, we report on the formation of 2D materials using boron cluster compounds. Through low-energy electron irradiation of self-assembled monolayers (SAMs) of carborane molecules, we achieved covalent lateral cross-linking, a process we were able to follow using scanning tunneling microscopy (STM) at unprecedented levels of detail, even between irradiation steps. Our research details the complete process, starting with the formation of highly crystalline SAMs from two specific carborane molecules, **O9,12** and **1-HS-bis-*p*CB**, deposited *via* vapor deposition on a Ag(111) substrate. Initially, these molecules assemble into periodic hexagonal arrays as analyzed using STM and LEED. Upon exposure to low-energy electron irradiation, these SAMs undergo cross-linking to form robust covalent networks. This cross-linking was investigated using complementary spectroscopic and microscopic techniques including XPS, UPS, STM, LEED and ToF-SIMS. We analyze the structural modifications during electron irradiation in detail and correlate them to possible changes in the molecular structure using DFT calculations. Especially the stepwise STM analysis shows the material conversion in high-resolution tracking the transition from the originally long-range order SAM to the formation of mechanically stable nanomembranes. In the final step, we confirm the high quality of the formed 2D-material by transferring them onto new substrates including the successful free-standing formation on TEM grids.

This study not only demonstrates the feasibility of using carborane SAMs as precursors for the tailored synthesis of 2D boron-rich materials but also introduces a methodology for observation of the cross-linking process. The implications of our findings extend to a wide range of borane and heteroborane molecules, facilitating the exploration of pristine borane layers,^48^ and enabling the achievement and study of homogeneous doping in these membranes using heteroborane constituents with a broad range of elements from the p- or d-blocks of the periodic table.^49−51^ The potential applications of these materials are vast, including their use in electronics due to their aromaticity, in catalysis thanks to the incorporation of catalytically active metal atoms, and as intermediates toward long-range borophene-like structures. Our study paves the way to a wide variety of future experiments, paralleling those conducted with carbon-based analogues, and sets the stage for discovering the full potential of these materials in various advanced technological fields.

## Methods and Experimental Details

### Sample Preparation

**O9,12** was synthesized using the method described in the literature.^34,44^

#### Preparation of **1-HS-bis-*p*CB**

A hexane solution of *n*-BuLi (1.6 M, 2.97 mmol, 1.9 mL) was dropwise added (over a period of 10 min) to a solution of 1,1′-Bis(1,12-dicarba-*closo*-dodecaborane) (0.85 g, 2.97 mmol) in Et_2_O (100 mL) at 0 °C. The mixture was stirred for 2 h at room temperature and then cooled again to 0–5 °C in an ice–water bath. At this temperature, sulfur (0.095 g, 2.97 mmol) was added with vigorous stirring. The reaction mixture was allowed to warm to room temperature and further stirred another 20 h. After this time, the mixture was quenched with 10% HCl (100 mL), and the organic phase was separated. The water phase was additionally extracted with Et_2_O (2 × 50 mL). The combined Et_2_O fractions were dried with anhydrous MgSO_4_, and the solvent was evaporated under reduced pressure to yield 0.91 g of a light-yellow powder. The crude product was purified by sublimation at reduced pressure with a slow increase of the bath temperature from 80 to 120 °C. The **1-HS-bis-*p*CB** (0.39 g, 41%) was collected at 120 °C as a white powder. Successful synthesis was verified via nuclear magnetic resonance spectroscopy (^11^B and ^1^H NMR, see Figures S14, S15).

The starting precursor for the preparation of **1-HS-bis-*p*CB**, 1,1′-Bis(1,12-dicarba-*closo*-dodecaborane), was prepared according to the literature.^52^

The SAMs were prepared by evaporation in the same UHV system (<2 × 10^–10^ mbar) used for XPS, UPS, STM, and LEED measurements using a molecular evaporator (Kentax). The **O9,12** molecules were evaporated at 70 °C and the **1-HS-bis-*p*CB** at 90 °C for 1 h on 300 nm Ag/mica substrates (Georg Albert PVD) or silver single crystal (Ag(111), Matek, purity 99,999%) respectively, which were held at room temperature. The substrates were cleaned before by repeated sputtering with Ar^+^ (1 keV, 10 mA) and annealing at 370 °C (more information on the cleaning process can be found in ref (18)).

### Characterization: NMR, MS, IR, Computational Optimization

NMR spectra were recorded on a JEOL ECZ 600 R NMR spectrometer. Chemical shifts were analyzed with reference to the solvent (δ = 7.26 ppm, CDCl_3_, 600 MHz, 295 K) and boron (δ = 0 ppm, relative to BF_3_(OEt)_2_) in CDCl_3_, 192.6 MHz, 295 K for the ^1^H and ^11^B{^1^H} NMR spectra, respectively.

The electron irradiation took place in the same UHV system used for the XPS measurements. The samples were irradiated at 50 eV and at different electron doses up to 50 mC/cm^2^ using a NEK 150SC electron gun (Staib).

Annealing of the cross-linked **O9,12** samples was conducted in the same UHV system on a heatable manipulator with a pyrolytic boron nitride (PBN) resistive heater placed below the sample. The temperature of the sample was measured by using a thermocouple. For temperatures above 400 °C additionally, a temperature control on the molybdenum sample holder using a two-color pyrometer (SensorTherm) with the emissivity coefficients of 23% was employed. Heating of the samples to the target temperature was achieved with a rate of 1–2 °C/min and kept constant for 30 min before cooling down. Samples prepared on Ag/Mica were annealed up to 400 °C, and for higher temperatures, the Ag(111) single was used as substrate.

After irradiation, the nanomembranes were transferred onto silicon wafers with an oxide surface layer (300 nm, Sil’tronix) and onto TEM grids (Quantifoil R 0.6/1). To transfer the nanomembrane, a 70 nm thick poly(methyl methacrylate) (PMMA) layer was applied to the surface. For this purpose, PMMA (50K, AR-P 631.04) was spin-coated onto the nanomembrane while it was still on the silver substrate and heated for 5 min at 50 °C on a hot plate. The second layer of PMMA (950 K, AR-P 671.04) was then applied under the same conditions and baked for 30 min at 50 °C.

To separate the silver substrate from the nanomembrane, the sample was placed on the surface of an iodine–potassium iodide solution (I_2_/ KI/H_2_O in a mass ratio of 1:4:10) for 24 h. The iodine reacts with silver to form silver(I) iodide, which dissolves in aqueous solution. Thus, the silver substrate deposited on mica can be separated from the nanomembrane with PMMA on top. Subsequently, excess iodine was reduced with a sodium thiosulfate solution. The samples were then repeatedly washed with ultrapure water (0.056 μS, MembraPure Aquinity2 E35), transferred onto the silicon wafer or TEM mesh, dried for 4 h at 50 °C, and used for further analysis.

To remove the PMMA layer after transfer, the samples on the silicon wafers were immersed in acetone for 20 min, rinsed with isopropanol, and dried under a stream of nitrogen. To minimize any damage to the free-standing membranes, supercritical drying (Autosamdri-815, Tousimis) was performed for the samples on TEM meshes.

The solvents 2-propanol (H_2_O ≤ 0.1%, purity ≥99.8%), acetone (H_2_O ≤ 0.2%, purity ≥99.8%), and ethanol (H_2_O ≤ 0.2%, purity ≥99.8%) were obtained from VWR Chemicals and used as delivered.

### Measurements

XPS and UPS were measured by using a UHV Multiprobe system (Scienta Omicron) with a monochromatic X-ray source (Al K_α_, 1486.7 eV) and a gas discharge He I vacuum light source (21.22 eV). The electron analyzer (Argus CU) has a spectral energy resolution of 0.6 eV (XPS) and 0.1 eV (UPS), respectively. The pass energies of 50 eV for the survey XP spectra, 30 eV for high-resolution XP spectra and 5.5 eV for UP spectra were used. The XP spectra were calibrated by referencing the binding energy of the Ag 3d_5/2_ signal at 368.2 eV and fitted using Voigt functions (30:70) after linear background subtraction. The energy scale in the UP spectra is referenced to the Fermi edge of the Ag substrate. Calculations of stoichiometry were performed with the software CasaXPS using the relative sensitivity factors of 1.68 (S 2p), 0.49 (B 1s), and 1.0 (C 1s); layer thickness was calculated using the Lambert–Beer equation. A mean free path of 27 Å^53,54^ was used for electrons that were released from the silver substrate and reached the detector through the SAM. The analyzer’s acceptance angle α was 19°. The layer thickness and error bar, corresponding to a 95% confidence interval, were calculated from the mean value of ∼10 samples. From UP spectra, the work function was determined by analyzing the secondary electron cutoff and the Fermi edge of the Ag substrate after applying a bias voltage of 10 V.

Optical microscopy was performed in bright-field mode with a Zeiss Axio Imager Z1.m microscope. The microscope was equipped with a thermoelectrically cooled 3 megapixel CCD camera (Axiocam 503 color).

The SEM images were obtained with a Zeiss Sigma VPfield emission scanning electron microscope at a beam power of 10 kV using the system’s in-lens detector.

LEED was performed with a single microchannel plate (SMCP)-LEED (Scienta Omicron) at a filament current of 0.8 A and a beam current of 2 nA with beam energies between 10 and 150 eV. Data acquisition was performed using Scienta Omicron’s LEEDControl software. For the quantitative LEED analysis, we corrected the original images from distortion with the LEEDCal software.^55,56^ The distortion-free LEED images were analyzed by fitting the visible LEED spots and simulating of the received structures using LEEDLab software.^55,56^

STM was performed in UHV at room temperature using VT SPM (Scienta Omicron) and an etched W tip, which was sputtered with Ar^+^ before use. Typical parameters for the bias voltage and tunneling current were 0.1 V and 1.0–1.5 nA, respectively. The evaluation of the STM images was carried out with the software Gwyddion.

Mass spectrometry was measured using a Pfeiffer PrismaPro QMG 250 F3 mass spectrometer during the evaporation of the **O9,12** molecule.

## Supporting Information Available

Mass spectrum of **O9,12** molecules and ToF-SIMS data of **O9,12** SAM, additional XPS data as well as table of peak assignments, additional STM/LEED images and analysis, detailed DFT computational investigation, UP spectra, NMR spectra of **1-HS-bis-***p***CB**. (PDF)
