# Supplementary material for: Carborane Nanomembranes
Source: ACS Nano. 2025 Feb 19;19(8):8131–41. doi: 10.1021/acsnano.4c16611 (PMC11887487; doi:10.1021/acsnano.4c16611)
Supplement: Supplementary file 1 — nn4c16611_si_001.pdf [file nn4c16611_si_001.pdf]

Supporting Information

Carborane Nanomembranes

Martha Frey,[1] Julian Picker,[1] Christof Neumann,[1] Jakub Višňák,[2, 3] Jan Macháček,[2] Oleg L. Tok,[2] Petr Bábor,[4] Tomas Base,[2] Andrey Turchanin[1, 5, 6*]*

[1]Friedrich Schiller University Jena, Institute of Physical Chemistry, Lessingstraße 10, 07743 Jena, Germany

[2]The Czech Academy of Sciences, Institute of Inorganic Chemistry, 250 68 Husinec-Rez, 1001, Czech Republic

[3]Department of Chemistry, Middle East Technical University, Ankara 06800, Türkiye

[4]Central European Institute of Technology (CEITEC), Purkyňova 123, 612 00 Brno-Královo Pole, Czech Republic

[5]Center for Energy and Environmental Chemistry Jena (CEEC Jena), Philosophenweg 7a, 07743 Jena, Germany

[6]Jena Center for Soft Matter (JCSM), Philosophenweg 7, 07743 Jena, Germany

KEYWORDS carboranes, two-dimensional materials, molecular self-assembly, electron irradiation induced chemical synthesis, nanomembranes

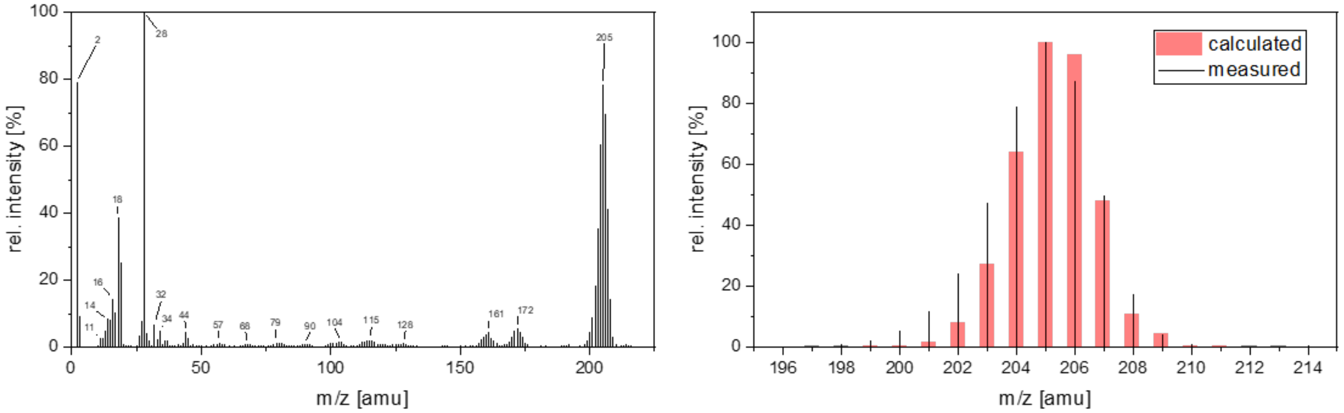

Figure S1. a) Mass spectrum of **O9,12**. b) Measured and calculated isotopic distribution envelopes for molecular masses corresponding to [M - 3H]- (M: $C_2B_{10}H_{12}S_2$).

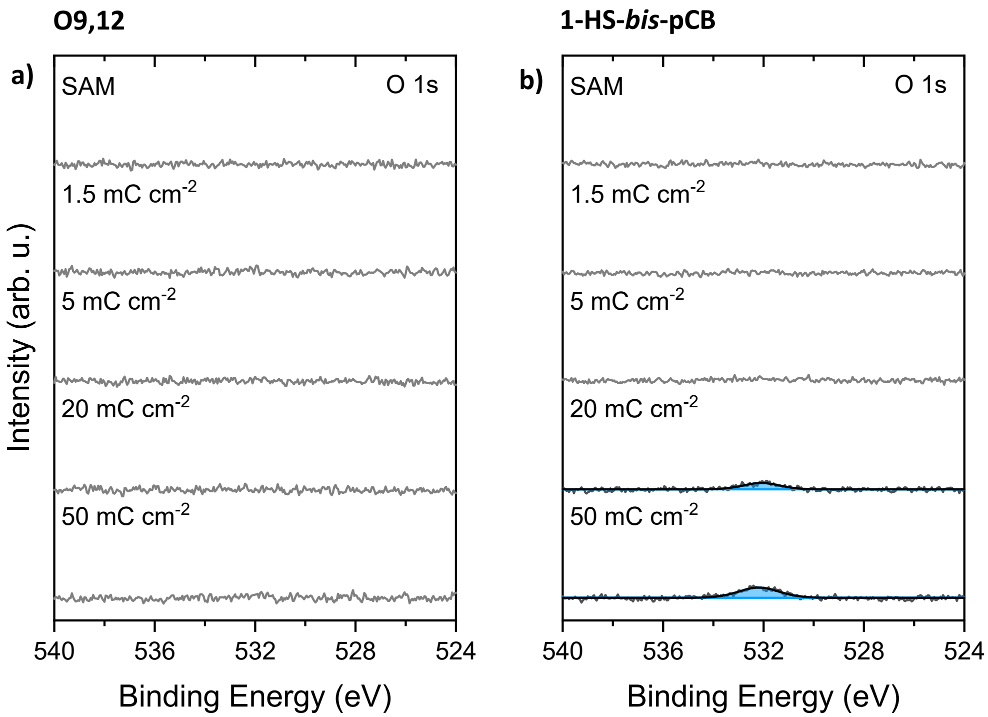

Figure S2. O 1s XP spectra of a) **O9,12** and b) **1-HS-bis-*p*CB** SAMs, stepwise cross-linked into a nano-membrane *via* electron irradiation with an energy of 50 eV.

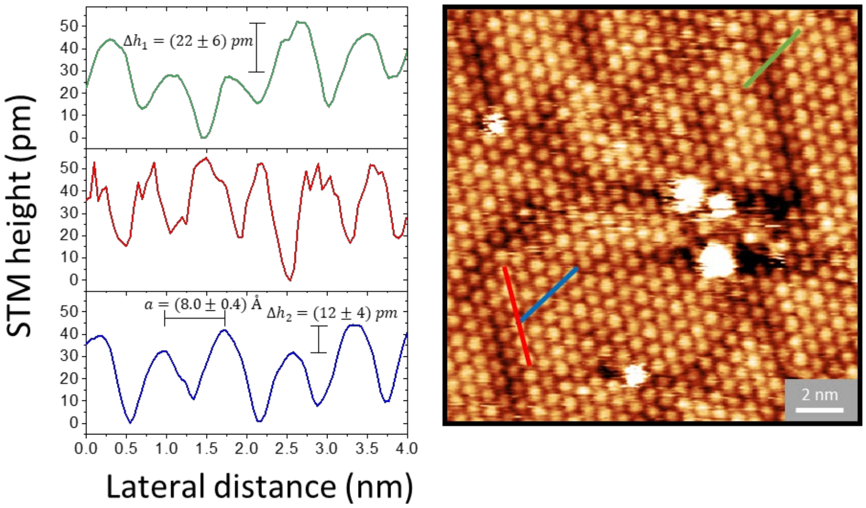

Figure S3. STM height profiles along the colored lines in the corresponding STM image ($20 \times 20\ nm^2$) of the **O9,12** SAMs on Ag(111). From all three height profiles we could determine the distance between the molecules a to be (8.0 ± 0.4) Å. This value is in good agreement with the lattice vectors of the one molecule structure (green lattice in Figure 3a). Additionally, the green line clearly shows two different molecule configurations with a STM height difference Δh_1 of (22 ± 6) pm. Both configurations could be explained with monovalent (lower STM height) or divalent bound molecules to the Ag(111) substrate. Although the geometrical height of the monovalent molecule is higher than the height for the divalent molecule, the opposite is true for the STM height according to the theoretical calculation of these **O9,12** carboranes on Au(111)[1]. Thus, we can confirm, besides XPS, also with STM, that the divalent configuration is predominant (Figure 3a). Furthermore, we see a height alternation of the molecules (Δh_2 of (12 ± 4) pm) along the blue line, which is not observed along the other lattice vector direction (red). Because of these observations we can confirm the blue lattice in Figure 3c, whereby the unit cell consists of two molecules and the second lattice vector is twice as long as the other.

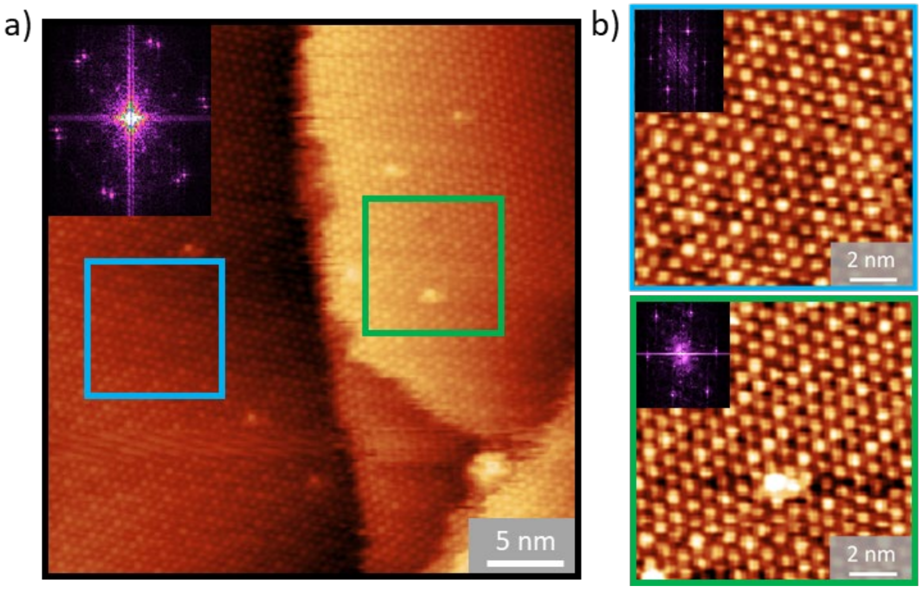

Figure S4. a) Large-area (35 × 35 nm^2) STM image of **1-HS-bis-*p*CB**. In b) two mirror domains that were cropped from a) are shown. The corresponding FFTs of the two mirror domains are shown as insets. The hexagonal spots of both mirror domains are rotated by approximately 6° in agreement with LEED results (6.0(2)°; blue structure in Figure 3). (STM conditions: 0.5 nA, 1.4 V, 293 K).

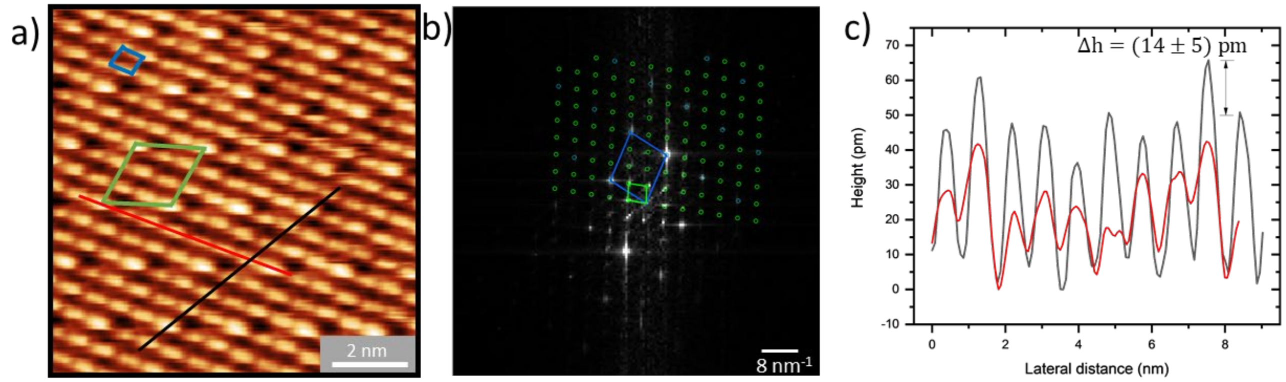

Figure S5 a) 10 × 10 nm^2 STM image of a **1-HS-bis-*p*CB** monolayer on Ag(111) where two different unit cells are shown in blue and green. b) The corresponding FFT image. The same unit cells are also shown in this FFT image. Both structures could be fitted to the visible spots and correspond to the **1-HS-bis-*p*CB** structure (blue; see Figure 3b, Table 1) and a commensurate supercell (green). The supercell consists of 7 carborane molecules, thereby the bright molecules in a) are sitting in top position related to Ag(111) surface, the others do not (see Figure S3) The STM height difference calculated from the line scans are measured to be (14 ± 5) pm. The following equation shows the fitted epitaxy matrix for the relation between the green and blue structure:

$$\begin{pmatrix} \vec{a_1} \\ \vec{a_2} \end{pmatrix} = \begin{pmatrix} 2.00(3) & -1.01(3) \\ 0.99(3) & 3.00(2) \end{pmatrix} \cdot \begin{pmatrix} \vec{b_1} \\ \vec{b_2} \end{pmatrix}$$

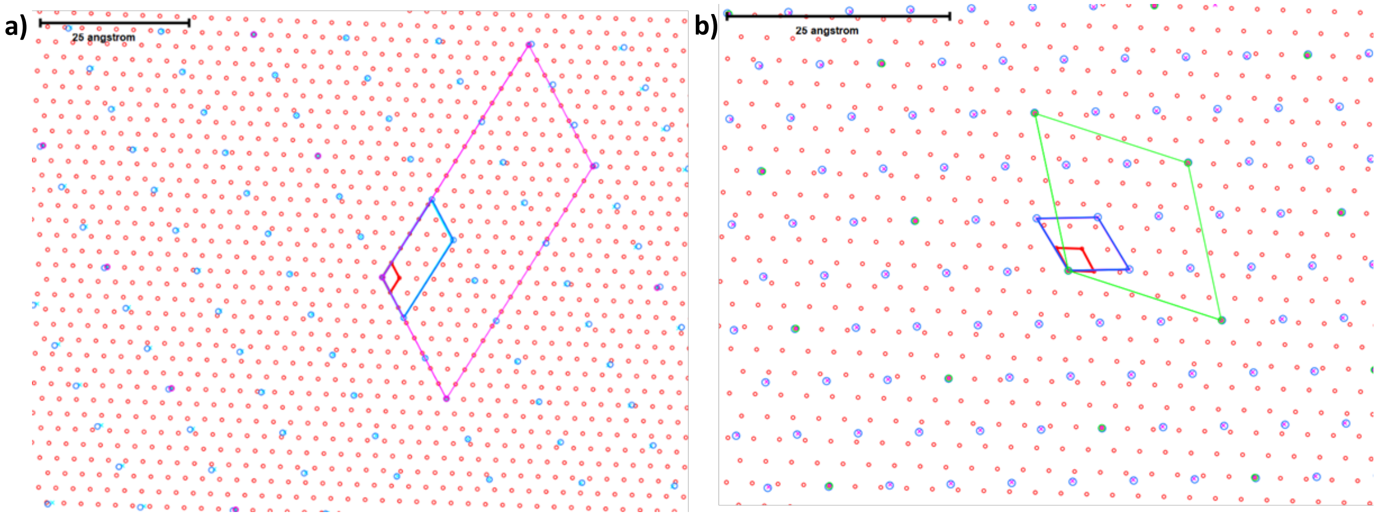

Figure S6. Real space representations of the lattice of a) **O9,12** and b) **1-HS-bis-*p*CB** monolayers on Ag(111). In a), the red circles, the blue circles, the cyan crosses, and the violet circles correspond to the Ag(111) lattice, the fitted **O9,12** lattice (two molecules), a perfect HOC lattice which is close to the fitted **O9,12** lattice, as well as the corresponding commensurate supercell of the HOC lattice. In b), the red circles, the blue circles, the violet crosses, and the green circles represent the Ag(111) lattice, the fitted **1-HS-bis-*p*CB** lattice, a perfect HOC lattice which is close to the fitted **1-HS-bis-*p*CB** lattice, as well as the corresponding commensurate supercell of the HOC lattice. The following equation shows the epitaxy matrix for the relation between the green and blue structure:

$$\begin{pmatrix} \overrightarrow{a_1} \\ \overrightarrow{a_2} \end{pmatrix} = \begin{pmatrix} 2.00 & -1.00 \\ 1.00 & 3.00 \end{pmatrix} \cdot \begin{pmatrix} \overrightarrow{b_1} \\ \overrightarrow{b_2} \end{pmatrix}$$

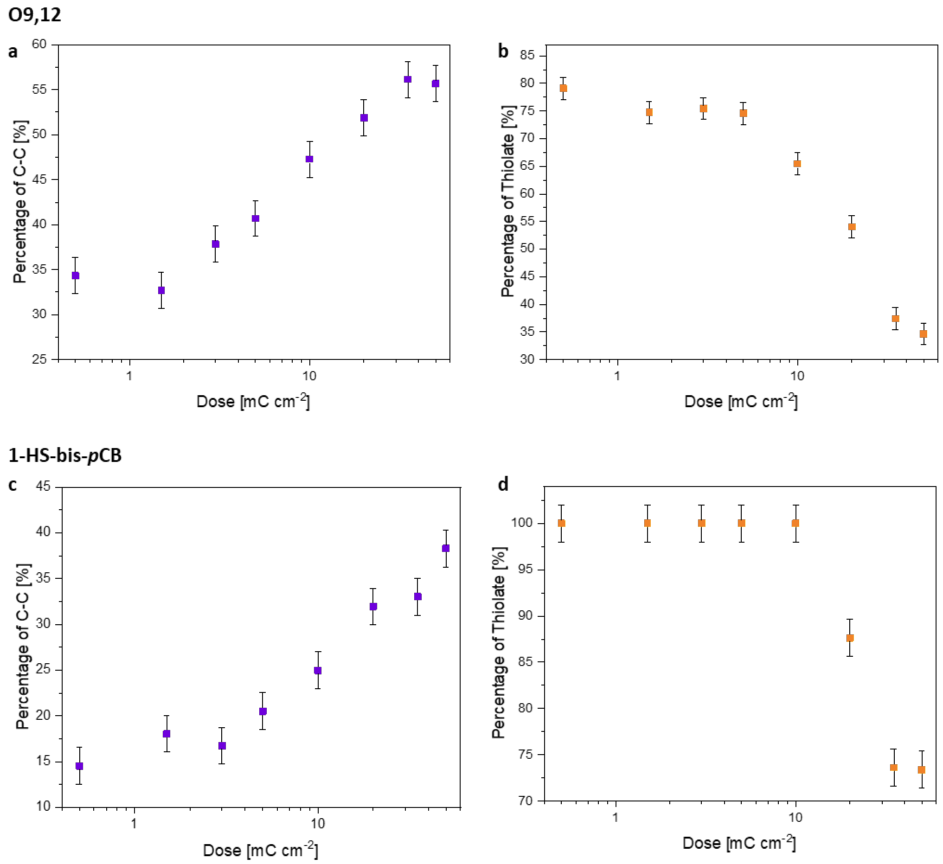

Figure S7. a), c): Percentage of carbon species with a binding energy of 284.8 eV commonly attributed to carbon-carbon bonds in the total amount of carbon depending on irradiation dose. b), d): The percentage of the sulfur component with a binding energy of 161.4 eV corresponds to thiolate bonds in total sulfur depending on the irradiation dose. a), b) Shows data of the cross-linking process of a **O9,12** SAM and c), d) a **1-HS-bis-*p*CB** SAM.

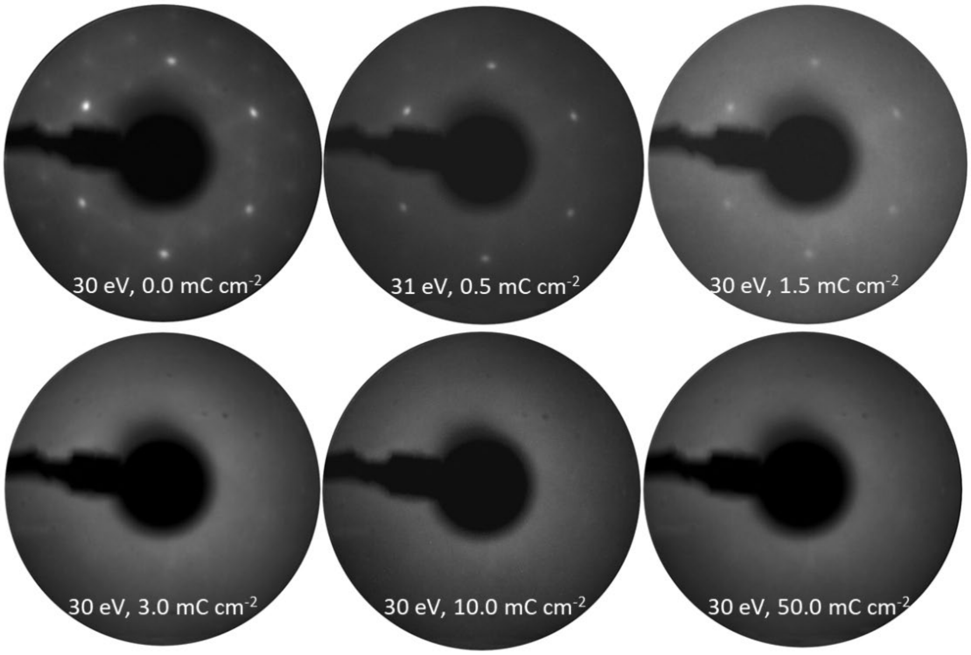

Figure S8. LEED patterns of **O9,12** SAMs on Ag(111) for different electron irradiation steps, whereby doses and the used electron energy of the LEED measurements are written in the respective images. Due to electron irradiation, the molecules are cross-linked with each other and thus lose their long-range ordered structure. This behavior can be observed using LEED, as the intensity of the most intense LEED spots decreases with increasing irradiation dose and vanishes above 3.0 mC cm^{-2}. The other LEED spots disappear almost entirely after the first irradiation step of 0.5 mC cm^{-2}. The order decrease for higher electron dose could also be measured with STM (Figure 4).

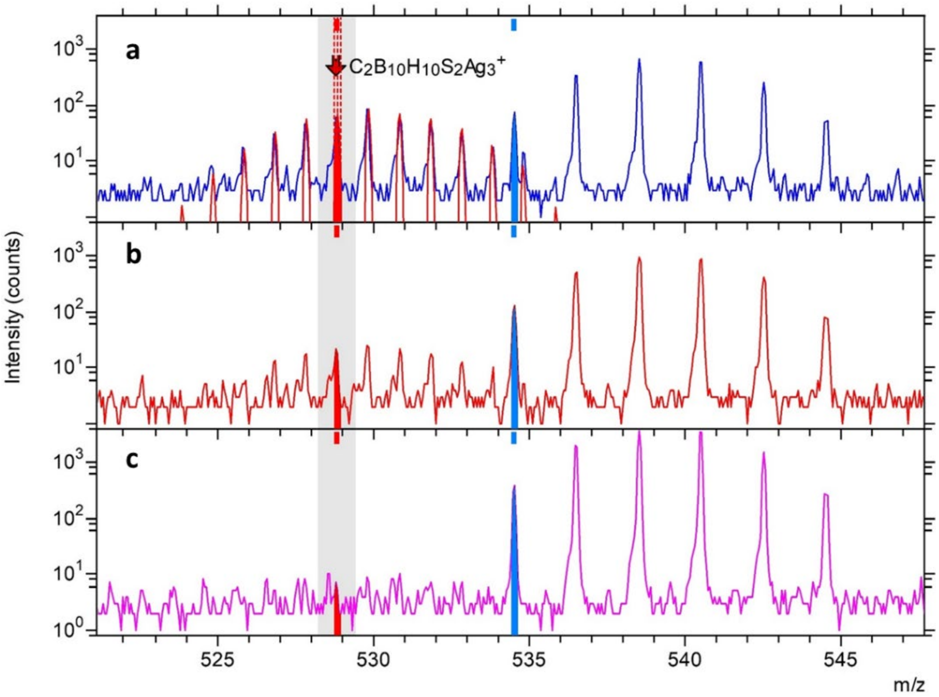

Figure S9. a) Time-of-Flight Secondary Ion Mass Spectrum of **O9,12** SAM on a silver surface with the characteristic mass fragment of M_1Ag_3 at 530 MU (positive polarity), M: $C_2B_{10}H_{10}S_2$. b), c) Continuous disappearance of the characteristic fragment as a consequence of electron irradiation with doses.

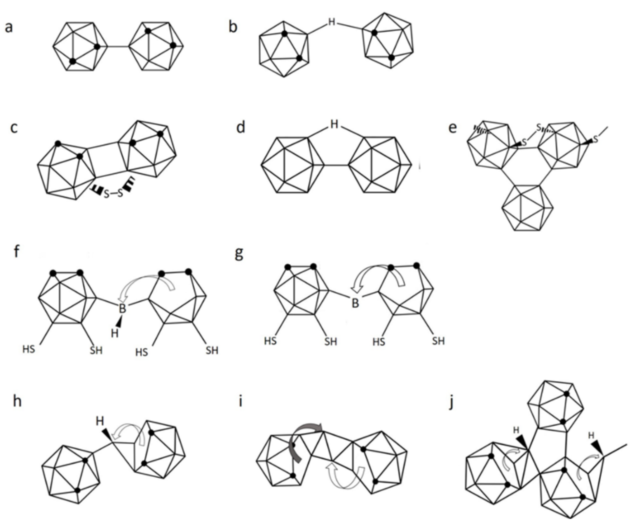

Figure S10. Schematic representation of computationally investigated structural features in crosslinked **O9,12** carborane monolayer. a) Single vertex-vertex bond/connection (top view), b) μ-H bridging hydrogen atom (top view), c, d, e) four-, five-, and six-membered rings with disulfide bonds depicted in c) and e), f, g) μ-BH and μ-B bridging moieties originating in one of the two equivalent vertices (3,6) of the carborane cages, h) BH vertex pulled slightly out of the carborane cage into the bridging position, i) two vertices (3,6) ripped out of the original carborane frameworks and turned into a triangular bridge, j) a structure depicting BH vertices and their role in crosslinking multiple molecules into a 2D network.

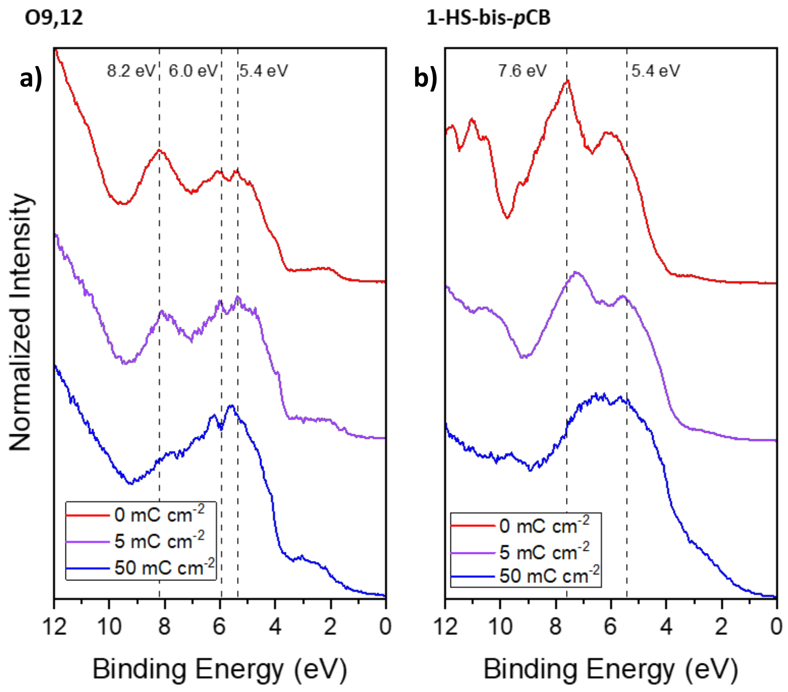

Figure S11. UP spectra for SAM, partially cross-linked SAM, and cross-linked SAM of a) **O9,12** and b) **1-HS-bis-*p*CB** on Ag/Mica substrate.

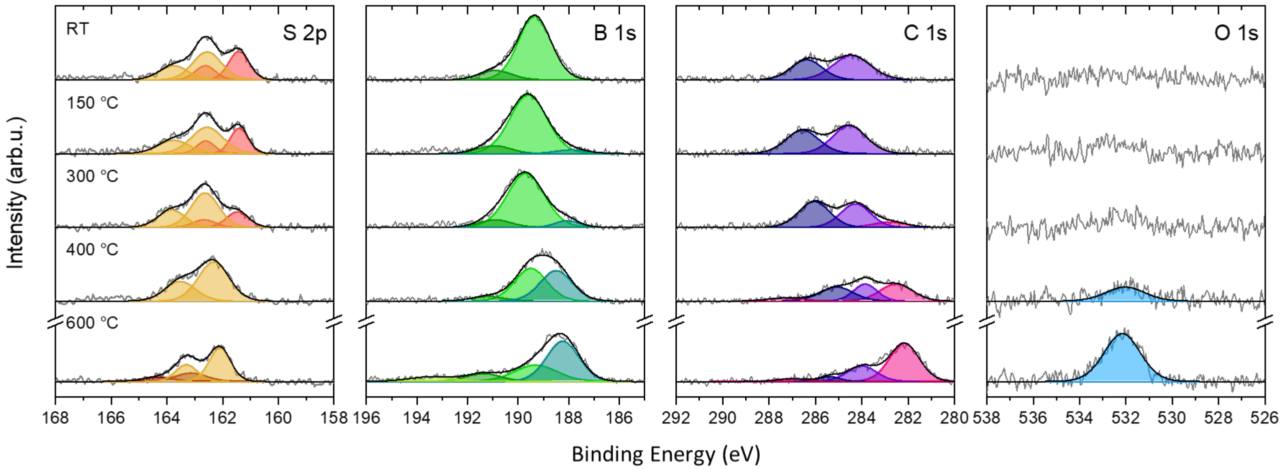

Figure S12. Room temperature, high resolution S 2p, B 1s, C 1s and O 1s XP spectra of cross-linked **O9,12** samples on Ag/Mica (RT – 400 °C) and Ag(111) (600 °C), respectively, after different annealing steps. The results confirm that the samples are thermally stable up to ~300 ° C. Between 300 – 400 °C, new C-B bonds are formed, as seen by a new component in the C 1s spectrum at ~282 eV, and the boron peak shifts to lower binding energies, which proceeds further at higher temperatures. Annealing to higher temperatures results also in desorption of sulfur and boron atoms, whereas despite the UHV conditions, carbon intensity increases and additional oxygen species appear in the spectrum. We point out that the samples still consist mostly of boron atoms and the adsorbed carbon impurities make up roughly 10% of total carbon based on the increase in area of the carbon peak. The C/S/O/B ratios of the annealed samples are (1.8±0.4) : (0.9±0.2) : 0 : 5 for 300 °C and (1.8±0.4) : (0.7±0.1) : (0.7±0.1) : 5 for 600 °C.

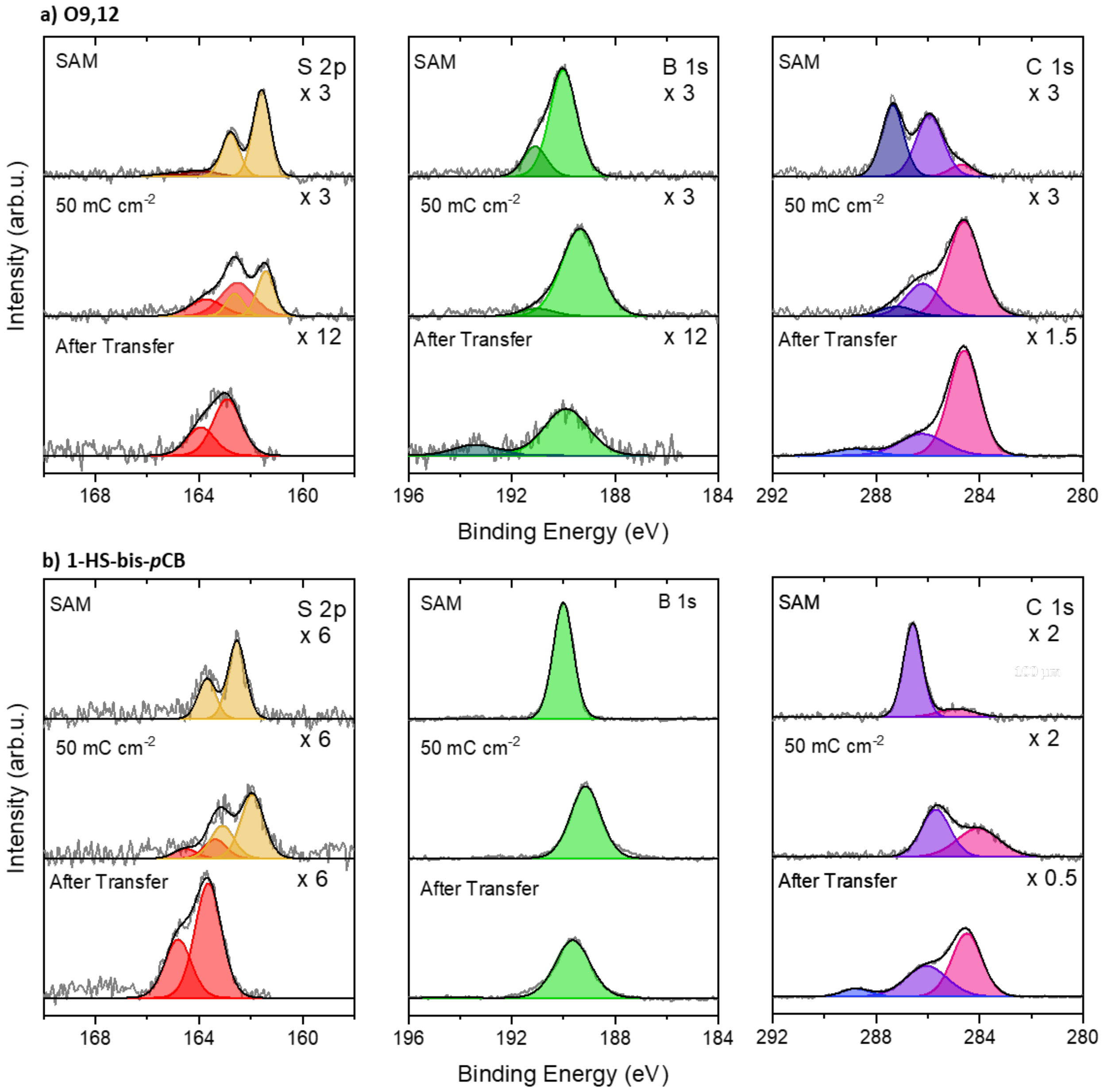

Figure S13. S 2p, C 1s, and B 1s XP spectra of a) **O9,12** and b) **1-HS-bis-*p*CB** SAMs, stepwise cross-linked into a nanomembrane *via* electron irradiation with an energy of 50 eV and transferred *via* chemical etching method on a silicon/ silicon oxide wafer and TEM grids. The spectra's intensities have been multiplied by the indicated factor for better representation.

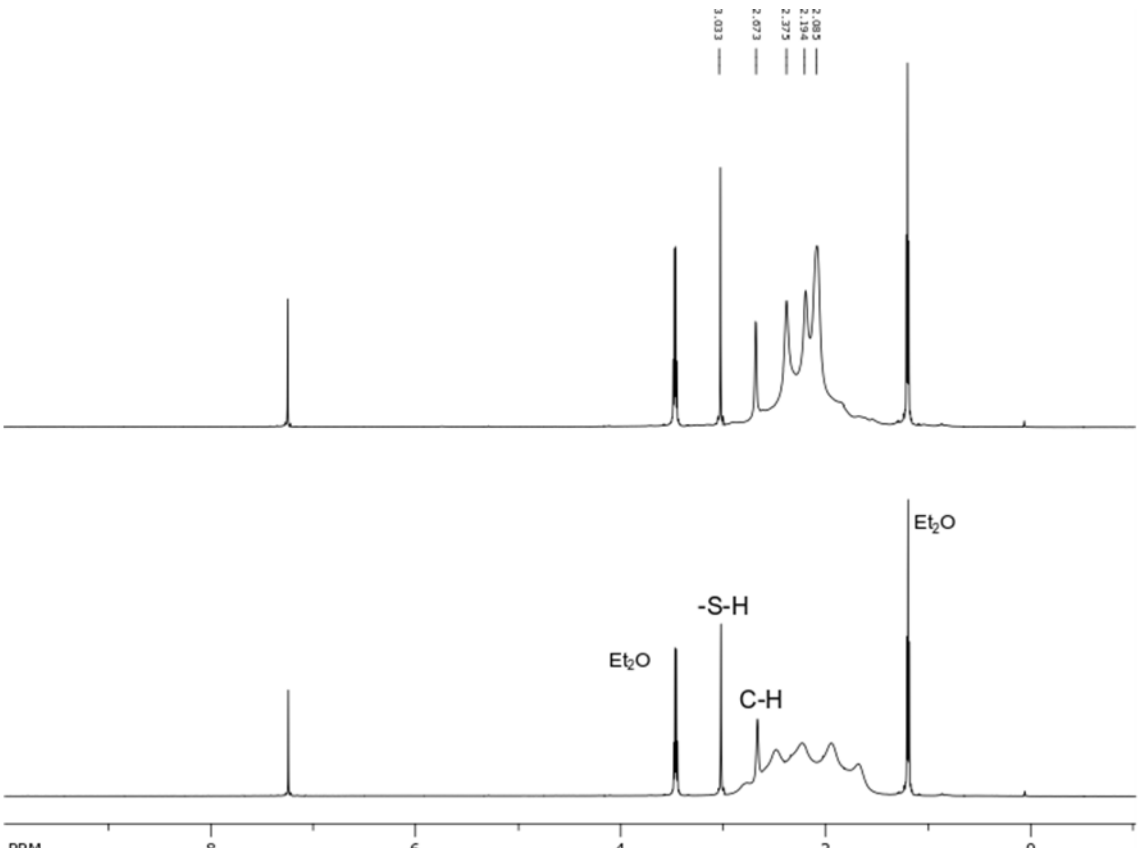

Figure S14. ^{1}H (bottom) and $^{1}H\{^{11}B\}$ (top) NMR spectra (600 MHZ, $CDCl_3$) of **1-HS-bis-*p*CB.**

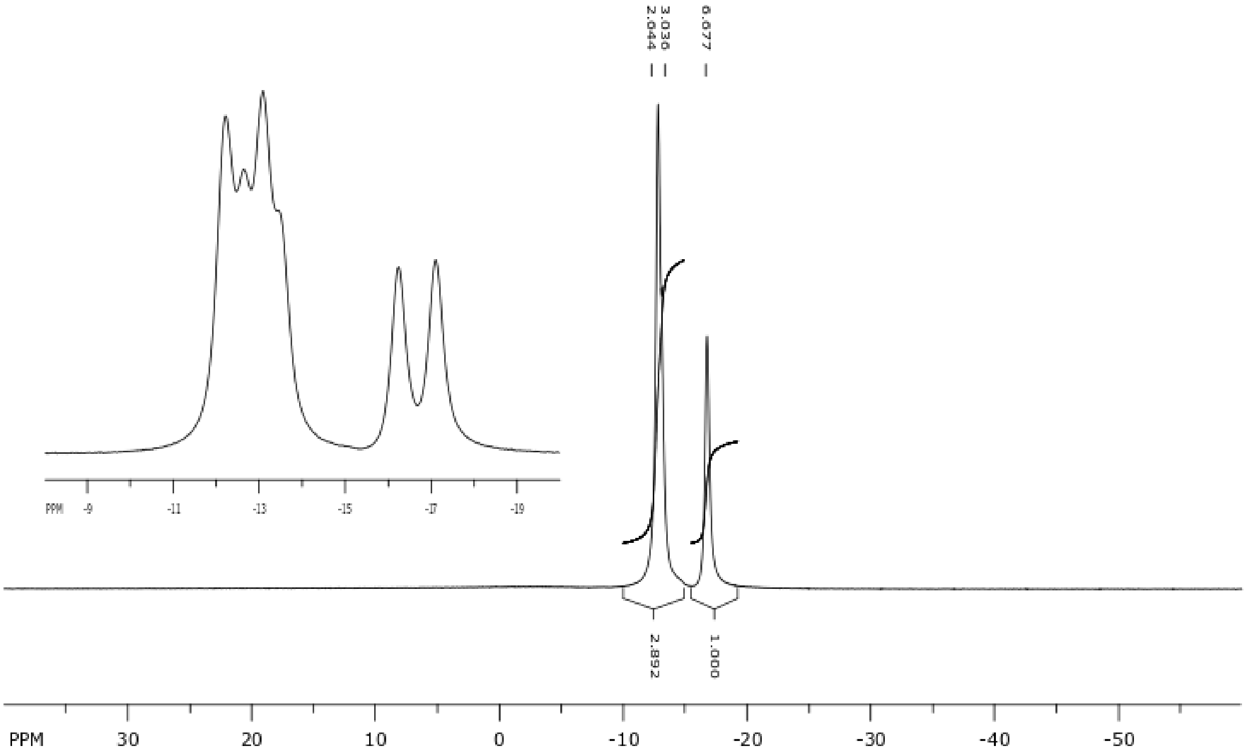

Figure S15. $^{11}B\{^{1}H\}$ and ^{11}B (expansion) NMR spectra (192.6 MHz, $CDCl_3$) of **1-HS-bis-*p*CB.**

Table S1. O9,12 peak assignment

Peak assignment	Binding energy, eV	fwhm, eV	Area, %
**C 1s**			
**SAM**			
C-C	284.8	2.1	28
carborane C-B	287.4	0.8	72
**Irradiated 1.5 mC/cm^2**			
C-C	285.0	1.7	33
carborane C-B	287.3	0.9	67
**Irradiated 5 mC/cm^2**			
C-C	284.9	1.5	41
carborane C-B	287.1	1.1	59
**Irradiated 20 mC/cm^2**			
C-C	284.6	1.5	52
carborane C-B	286.5	1.6	48
**Irradiated 50 mC/cm^2**			
C-C	284.3	1.5	56
carborane C-B	286.2	1.8	44
**S 2p$_{3/2}$**			
**SAM**			
thiolate	161.6	0.8	87
thiol/ disulfide	163.0	1.5	13
**Irradiated 1.5 mC/cm^2**			
thiolate	161.5	0.8	80
thiol/ disulfide	163.0	1.5	20
**Irradiated 5 mC/cm^2**			
thiolate	161.5	0.8	75
thiol/ disulfide	162.9	1.1	25
**Irradiated 20 mC/cm^2**			
thiolate	161.4	0.8	54
thiol/ disulfide	162.6	1.2	46
**Irradiated 50 mC/cm^2**			
thiolate	161.4	0.8	37
thiol/ disulfide	162.5	1.3	63
**B 1s**			
**SAM**			
carborane B	190.0	1.2	81
carborane S-B	191.1	0.9	19
**Irradiated 1.5 mC/cm^2**			
carborane B	190.0	1.3	84
carborane S-B	191.1	0.9	16
**Irradiated 5 mC/cm^2**			
carborane B	189.9	1.6	90
carborane S-B	191.0	1.5	10
**Irradiated 20 mC/cm^2**			
carborane B	189.5	1.7	93
carborane S-B	191.1	1.5	7
**Irradiated 50 mC/cm^2**			
carborane B	189.3	1.8	95
carborane S-B	191.1	1.5	5

Table S2. 1-HS-bis-*p*CB peak assignment

Peak assignment	Binding energy, eV	fwhm, eV	Area, %
**C 1s**			
**SAM**			
C-C	284.6	1.9	14
carborane C-B	286.5	0.9	86
**Irradiated 1.5 mC/cm^2**			
C-C	284.7	2.0	18
carborane C-B	286.3	1.0	82
**Irradiated 5 mC/cm^2**			
C-C	284.3	1.5	21
carborane C-B	286.1	1.1	79
**Irradiated 20 mC/cm^2**			
C-C	284.1	1.8	32
carborane C-B	285.8	1.3	68
**Irradiated 50 mC/cm^2**			
C-C	284.0	1.5	38
carborane C-B	285.6	1.4	62
**S $2p_{3/2}$**			
**SAM**			
thiolate	162.5	0.8	100
**Irradiated 1.5 mC/cm^2**			
thiolate	162.2	1.0	100
**Irradiated 5 mC/cm^2**			
thiolate	162.0	1.0	100
**Irradiated 20 mC/cm^2**			
thiolate	162.0	1.1	88
thiol/ disulfide	164.2	1.2	12
**Irradiated 50 mC/cm^2**			
thiolate	161.8	1.1	73
thiol/ disulfide	163.5	1.2	27
**B 1s**			
**SAM**			
carborane B and C-B	190.0	0.9	100
**Irradiated 1.5 mC/cm^2**			
carborane B and C-B	189.7	1.0	100
**Irradiated 5 mC/cm^2**			
carborane B and C-B	189.5	1.1	100
**Irradiated 20 mC/cm^2**			
carborane B and C-B	189.3	1.3	100
**Irradiated 50 mC/cm^2**			
carborane B and C-B	189.2	1.4	100

DFT computational investigation of crosslinking

O9,12

Generation of the Initial Configuration of O9,12 Molecules for DFT Optimization

Molecules of the studied carborane derivative (9,12-$(HS)_2$-1,2-$C_2B_{10}H_{10}$, referred to as O9,12), with either two thiol (-SH) anchoring groups, or with these being replaced by -H, -SAg, or -S^- (always specified in the text or by the resp. Table) were initially arranged in a hexagonal pattern depicted in *Figure S16*. This pattern has been used as a starting point to generate configurations of smaller subgroups of molecules for subsequent computational optimization. Fig. 1 also shows selection of these smaller subgroups containing two to six molecules (solid colored lines) in which we removed even number (2*n*) of hydrogen atoms (again, specified by each particular subgroup further in the sketches of initial computational setups with *n* red lines), thus creating new chemical bonds (*n*) cross-linking the molecules of the initial array. In order to promote bond formation and explore as wide variety of its modes as possible without actually modeling the real physical processes in detail, multiple starting geometries were generated from each subgroup by translating the individual molecules with respect to the geometric center of the subgroup of various fractions of their relative position, referred to as Parameter value in all the Tables. Negative values from -0.05 (-5 %) to -1.95 (an inverse position with distance reduced of 5 %) were tried; the direction of this translation is also depicted in the figures.

This computational investigation is aimed at providing first view on different local structural features that might form in the crosslinked carborane nanomembranes. It also shows a plethora of crosslinking possibilities resulting in many different local arrangements, which correspond to the loss of initial periodicity (characteristic of the starting self-assembled monolayer) observed as a consequence of electron-induced cross-linking.

Density Functional Theory (DFT)[1] with PBE functional[2] was used for the investigation with minimal basis set (single zeta Split Valence: SV[3-5], and for silver def2-SVP[6]). Multipole- accelerated Resolution of Identity with Coulomb fitting (MARIJ)[7] have been used to accelerate the computations. Geometry optimization was done using BFGS algorithm[8] and „relax“ method, both as implemented in Turbomole-7.1[9] software package. For each optimized structure, normal mode calculation has been done to assure that the energy minimum (and not a transition state) has been reached. All schematic images as well as optimized structures presented further in this Supporting Information were generated using Molden[10].

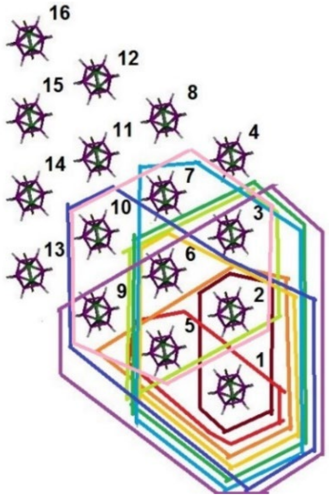

Figure S16. Initial hexagonal array of **O9,12** molecules showing the subgroups of 2 to 6 molecules (colored lines) for further computational analysis.

Results - Selected Structural Features

The following figure, Figure S17, shows a schematic representation of selected structural features commonly appearing in the optimized molecular structures addressed systematically within this computational investigation (see Computational Setups and Optimized Geometries below).

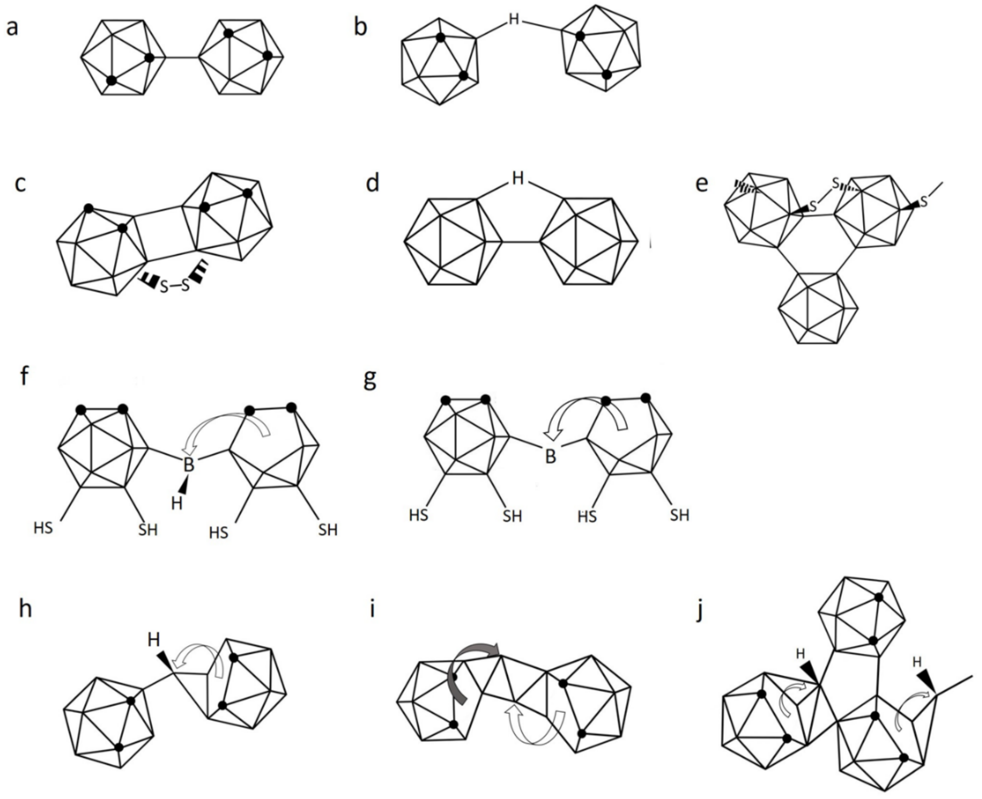

Figure S17. Schematic representation of typical structural features obtained by computational optimization in crosslinked **O9,12** carborane monolayers: a) Single vertex-vertex bond/connection (top view); b) μ-H bridging hydrogen atom (top view); c,d,e) four-, five-, and six-membered rings with disulfide bonds depicted in c) and e); f,g) μ-BH and μ-B bridging moieties originating from one of the vertices adjacent to the carbon atoms (black dots); h) BH vertex pulled slightly out of the carborane cage into the bridging position; i) two vertices (3 and 6) ripped out of the original carborane frameworks and turned into a triangular bridge; j) a structure depicting two BH vertices and the way they link multiple molecules.

1-HS-bis-*p*CB

Generation of the Initial Configuration of Molecules for DFT Optimization

All structures have been computationally optimised (and checked for imaginary frequency modes) using DFT[1] (PBE functional)[2], Multipole Accelerated Resolution of Identity with Coulomb Fitting (MARIJ)[7], BFGS energy minimiser[8] and using single zeta Split Valence (SV) basis set[3-5]. In some cases, specified in the text, more accurate def2-SVP (polarisation functions added to all atoms except hydrogen) and def2-TZVPP (triple zeta with polarization functions on all atoms) have been used[11]. Dispersion interactions have been accounted for using the empiric "D3" correction. Turbomole V7.1[9] has been used for computations and Molden for graphical outputs[10]. All computations have been done using MetaCentrum, a Czech computational grid.

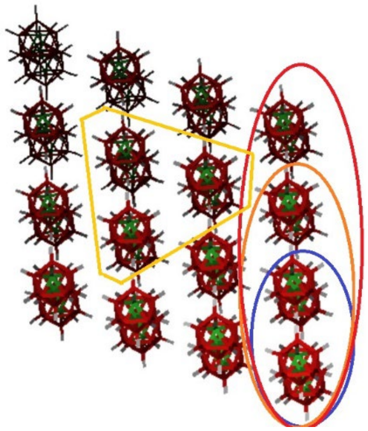

Figure S18. Initial array of 4 × 4 **1-HS-bis-pCB** molecules showing the selection of subgroups (coloured lines) for further computational analysis.

Results - Selected Structural Features

The following figure, *Figure S19*, shows a schematic representation of a few selected structural features commonly appearing in the optimized molecular structures. Many features are comparable to those observed in arrays of **O9,12** molecules.

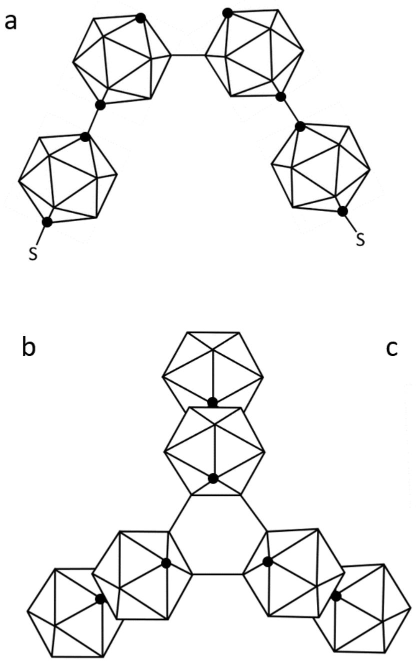

Figure S19. Schematic representation of two and three molecules of **1-HS-*bis*-pCB** linked by either single vertex-vertex bond(s) (*a)* side view of two molecules, *b)* top view of three tripod-like arranged molecules) or by a *c)* μ-BH bridging moiety which requires a vertex to be ripped out of the original cage.

Starting and Optimized Geometries

In the following section, an example of computational results is presented in the form of a Table of energies of the optimized structures, and the respective initial configurations shown below the Table. XYZ files of the optimized structures are provided separately.

Table S3. Energies and specification of the molecular configurations. Total number of molecules in the subgroup: 2, Anchoring group: SH, Minimum energy reference optimized structure (set as 0 in the Table): E = -2117.966960 Hartree atomic units (h.a.u.), Formula of the initial molecular configuration: 2 × $C_4B_{20}H_{19}SH$, Number of expected new intermolecular bonds in the optimized structure: 2.

xyz file number[a]	Molecular configuration[b]	Parameter value	E_rel [h.a.u.]	E_rel [eV]	E_rel [kcal/mol]	E_rel [kJ/mol]
1	B02.01	-0.40	1.8397	50.06	1154.4	4830.1
2[b] (def2SVP)	B02.01	-0.45	1.3558	36.89	850.8	3559.6
3[b] (def2TZVPP)	B02.01	-0.05	0.0000	0.00	0	0.0

[a] Number of the respective xyz file

[b] No intermolecular bonds formed, both molecules were too far from each other in the initial geometry

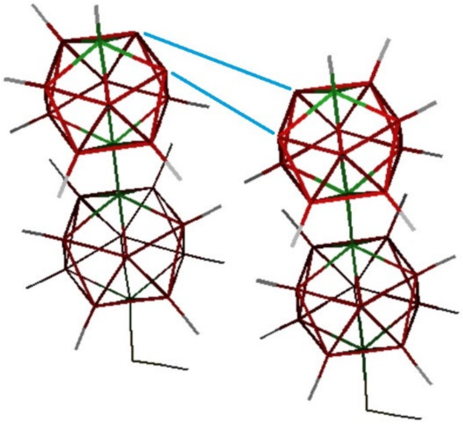

B02.01

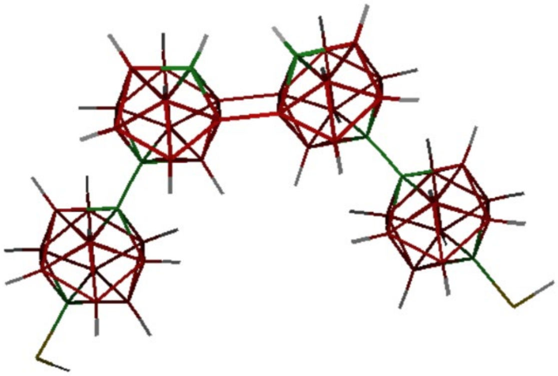

Optimized molecular structure (xyz file 1)

REFERENCES

1. Hohenberg, P.; Kohn, W., Inhomogeneous electron gas. *Physical Review* **1964,** *136* (3B), B864.

2. Ernzerhof, M.; Scuseria, G. E., Assessment of the Perdew–Burke–Ernzerhof exchange-correlation functional. *The Journal of Chemical Physics* **1999,** *110* (11), 5029-5036.

3. Schäfer, A.; Horn, H.; Ahlrichs, R., Fully optimized contracted Gaussian basis sets for atoms Li to Kr. *The Journal of chemical physics* **1992,** *97* (4), 2571-2577.

4. Stephens, P. J.; Devlin, F. J.; Chabalowski, C. F.; Frisch, M. J., Ab initio calculation of vibrational absorption and circular dichroism spectra using density functional force fields. *The Journal of Physical Chemistry* **1994,** *98* (45), 11623-11627.

5. Eichkorn, K.; Weigend, F.; Treutler, O.; Ahlrichs, R., Auxiliary basis sets for main row atoms and transition metals and their use to approximate Coulomb potentials. *Theor. Chem. Acc.* **1997,** *97*, 119-124.

6. Hellweg, A.; Rappoport, D., Development of new auxiliary basis functions of the Karlsruhe segmented contracted basis sets including diffuse basis functions (def2-SVPD, def2-TZVPPD, and def2-QVPPD) for RI-MP2 and RI-CC calculations. *Physical Chemistry Chemical Physics* **2015,** *17* (2), 1010-1017.

7. Sierka, M.; Hogekamp, A.; Ahlrichs, R., Fast evaluation of the Coulomb potential for electron densities using multipole accelerated resolution of identity approximation. *The Journal of Chemical Physics* **2003,** *118* (20), 9136-9148.

8. Fletcher, R., *Practical Methods of Optimization*. John Wiley & Sons: 2000.

9. Ahlrichs, R.; Armbruster, M.; Bachorz, R.; Bär, M.; Baron, H.; Bauernschmitt, R.; Bischoff, F.; Böcker, S.; Crawford, N.; Deglmann, P., TURBOMOLE 6.6. A development of University of Karlsruhe and Forschungszentrum Karlsruhe GmbH, 1989–2007, TURBOMOLE GmbH, since 2007, Karlsruhe, Germany. 2014.

10. Schaftenaar, G.; Noordik, J. H., Molden: a pre-and post-processing program for molecular and electronic structures. *J. Comput. Aided Mol. Des.* **2000,** *14*, 123-134.

11. Weigend, F.; Ahlrichs, R., Balanced basis sets of split valence, triple zeta valence and quadruple zeta valence quality for H to Rn: Design and assessment of accuracy. *Physical Chemistry Chemical Physics* **2005,** *7* (18), 3297-3305.
